# Harnessing Photochemistry in Natural Product Synthesis: From Strategy to Applications

**DOI:** 10.1021/acs.jnatprod.5c00874

**Published:** 2025-11-06

**Authors:** Elina K. Taskinen, Burkhard König

**Affiliations:** Department of Chemistry and Pharmacy, 9147 University of Regensburg, Universitätsstr. 31, 93053 Regensburg, Germany

## Abstract

Photochemistry and total synthesis are deeply rooted in the history of organic chemistry, each developing independently while also intersecting frequently. Indeed, mild reaction conditions, versatility of transformations, and complementary selectivities to thermal methods make photochemistry an especially powerful tool for the synthesis of complex target molecules. In this Review, we highlight recent examples of total syntheses (from 2020 to 2025) featuring photochemical reactions as pivotal steps. Although the application of photochemistry in total synthesis has been consistently reviewed throughout the past decades, we feel that the wider emergence of photocatalytic methods, together with the continued importance of certain direct irradiation approaches, warrants its own discussion. We hope that our analytical approach and strategic insights will help us to identify cases where photochemical reactions could prove useful, thereby further encouraging their use in total syntheses.

## Graphical abstract

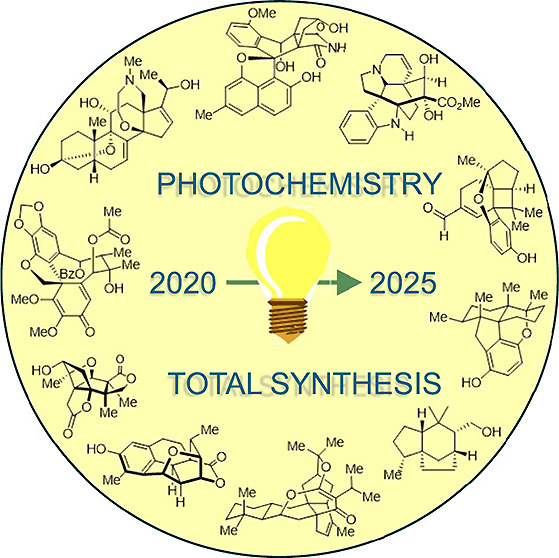

## 1 Introduction

It is safe to say that no other field has shaped chemistry as much as the total synthesis of natural products. For decades, relentless synthetic efforts toward naturally occurring structures have completely reshaped the ways chemists approach their synthetic targets,^1^ ignited the development of numerous novel transformations, and provided access to several medically endorsed molecules^2^ (Figure 1). Since the first realization of a synthetic process, the conversion of ammonium cyanate into urea in 1828,^3^ natural product synthesis has experienced several victories and brought about many Nobel-prize worthy discoveries.^4,5^ The beginning of the 20th century witnessed a significant leap as α-terpineol (Perkin, 1904),^6^ camphor (Komppa, 1903)^7^ and tropinone (Willstätter, 1901, and Robinson, 1917)^300,8^ were all successfully prepared in the laboratory, marking the advent of precise modification of aliphatic compounds. Moving toward the 1950s, advancements in structural analysis encouraged synthetic chemists to pursue increasingly complex target compounds. Some of the most famous accomplishments from this era include the total syntheses of (−)-strychnine and (−)-reserpine (Woodward, 1954 and 1958)^9,10^ as well as the synthesis of longifolene (Corey, 1961).^11^ Eventually, the quest toward ever more complicated natural products culminated in Kishi’s synthesis of palytoxin in 1994.^12,13^

**Figure 1:**
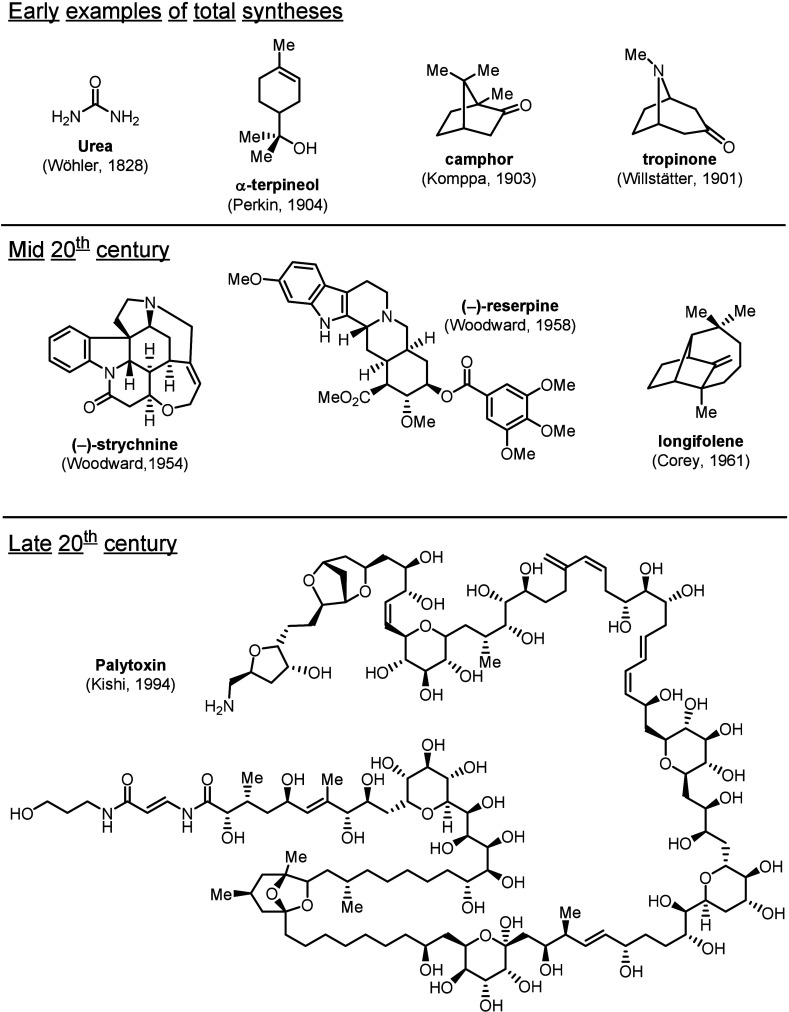
Landmarks of natural product total synthesis.

Since the turn of the century, the goals of total synthesis have shifted again.^14^ Instead of targeting the most complex (and synthetically demanding) structures possible, efficient and concise syntheses, sometimes as short as a few steps, have become more and more desirable.^15–17^ To achieve the complexity of natural products in such short sequences, convergent synthetic strategies have often been employed in combination with state-of-the-art methods for fragment couplings.^18^ Interestingly, in recent years, divergent syntheses have in turn gained more attention, resulting in syntheses of entire families of natural products *via* selective late-stage modification of a common precursor.^19,20^

Photochemistry, the field that focuses on light-driven transformations, has also a long and impressive history of its own. While the earliest applications of sunlight mainly focused on capturing its energy in the form of heat, over the years, scientists went on to observe notable changes upon the interaction of light with matter.^21^ More detailed accounts of the interaction of light with single molecules started to emerge at the end of the 19th century, which eventually led to the development of the flourishing field of photochemistry. While the past decades have been marked by notable advances in the field, photochemical reactions have played a vital part in enabling total syntheses for decades. For example, the final deprotection of the palytoxin carboxylate into the natural product was only possible by photochemical means.^13^ Taking into consideration the mild reaction conditions and high functional group tolerance of photochemical methods, it becomes clear why this contemporary synthetic field is so well suited for making natural products. In comparison with ionic methods, photochemistry often offers complementary selectivity for substrate activation (Figure 2A). Hence, functional groups, such as alkenes, carboxylic acids, hydroxy anions, and nitrogen-centered anions, can be activated by one-electron oxidations, thus creating electron-deficient radicals or radical cations as reactive intermediates. In contrast, one-electron reductions of functionalities such as aryl (pseudo)­halides, benzyl halides, alkyl iodides, α-halocarbonyls, and conjugated alkenes result in electron-rich open-shell species. Additionally, hydrogen atom abstraction (HAT) provides a facile way toward C–H activation on the hydridic positions. In this last case, the difference between acidic protons and hydridic protons is marked in blue and violet.

**Figure 2:**
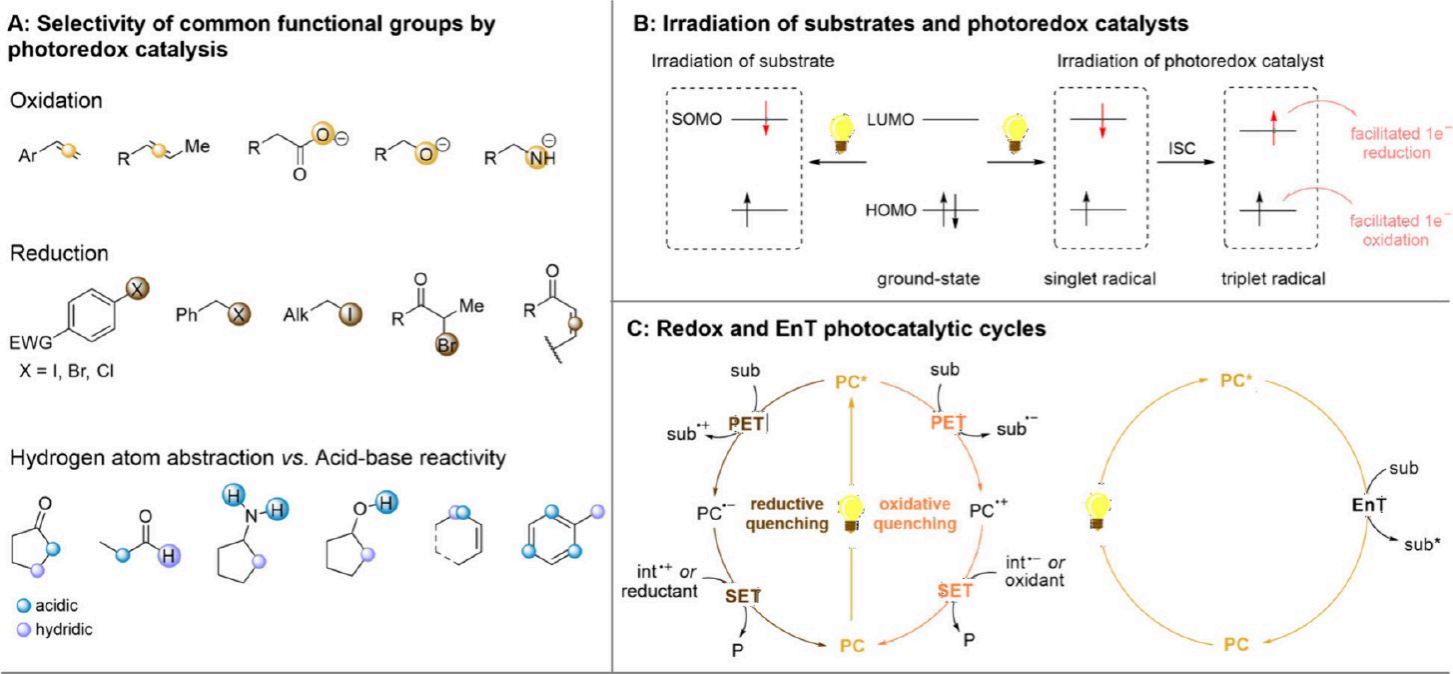
Functional group selectivity of photoredox catalysis (A), orbital representations of direct irradiation and photoredox catalysis (B), and general circles for photoredox and energy transfer catalysis (C).

Mechanistic and photophysical principles of photochemistry are summarized in Figure 2B. In general, photoredox catalysis can take place either *via* direct excitation of the substrate or *via* the intermediacy of a photocatalyst. In direct excitation methods, the substrate acts as a light-harvesting species, and light absorption promotes an electron from the highest occupied molecular orbital (HOMO) to the lowest unoccupied molecular orbital (LUMO), creating a reactive singly occupied molecular orbital (SOMO). Notably, the irradiation wavelength absorbed corresponds to the energy difference of the HOMO–LUMO gap. This electronic arrangement swiftly results in productive radical reactions or back-relaxation of the excited electron. In photocatalytic methods, however, the light irradiation is initially absorbed by the photocatalyst, in which one electron is again promoted to a higher energy orbital. This generates a singlet excited state, which can be converted to a slightly longer-lived triplet excited state *via* intersystem crossing (ISC). The excited-state catalyst, whether in its singlet or triplet state, is typically a better oxidant and a better reductant than the ground-state catalyst, and photoinduced electron transfer (PET) occurs upon engaging with a substrate. In a general photoredox catalytic cycle depicted in Figure 2C, one-electron oxidation of the catalyst often activates the ground-state substrate by generating a radical anion, whereas one-electron reduction of the catalyst yields a radical cationic intermediate from the substrate. For these reactivities, the reduction potential of the catalyst measures how easily the catalyst is reduced (while the substrate is oxidized), and the oxidation potential corresponds to one-electron oxidation of the catalyst (while the substrate is reduced). To close the catalytic cycle, a complementary single-electron transfer (SET) from a reaction intermediate or a terminal oxidant/reductant is required. In addition to electron transfer, the photocatalysts can also transfer their excitation to the substrate, upon which the catalyst is returned to its ground state, and the substrate is promoted to its excited state. In this direction, whether energy transfer (EnT) takes place is dependent on the excited state triplet energy of the catalyst and the triplet-state energy of the ground-state substrate.

For clarity, the structures of the photocatalysts discussed in this article are shown in Figure 3; they will be referred to by their names and acronyms later in the text. As a rule of thumb, the ruthenium and iridium photocatalysts are typically well-studied and robust under a variety of reaction conditions, overcoming the occasional challenges of degradation of organophotocatalysts. In turn, the addition of a charge to the organophotocatalysts is an efficient way to access highly oxidizing and highly reducing compounds, which might specify their means of substrate activation to either oxidation or reduction. Over the course of this review, some examples of the employment of less frequently used photocatalysts (such as FCNIrPic^+^ and Zn­(II)­porphyrin) are also encountered, demonstrating the broad variability of the photocatalytic species. While our goal in this article is to mainly discuss general concepts with some representative examples, more comprehensive reviews on the use of photochemistry in natural product synthesis can be found elsewhere.^22–27^

**Figure 3:**
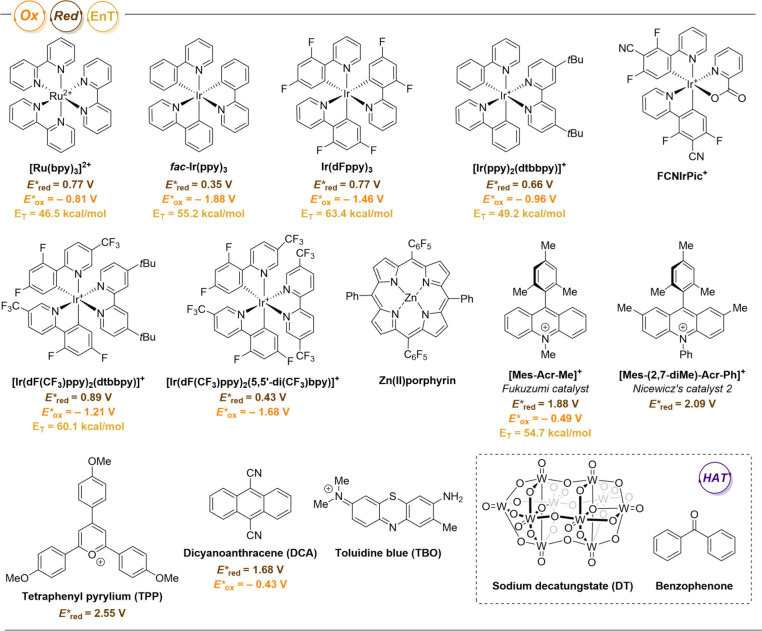
Photoredox catalysts discussed in this Review and their estimated excited-state redox potentials.

## 2 Application of Photochemistry in Total Synthesis

### 2.1 [2 + 2]-Cycloaddition

[2 + 2]-Cycloaddition, conversion of two alkenes into a cyclobutene moiety, is one of the classical examples of a photochemical reaction (Scheme 1, upper part). From a synthetic perspective, the reaction holds great importance, as it offers a mild and facile method for generating highly strained cyclobutane products. With its long-standing history, the [2 + 2]-cycloaddition together with similar Paternò–Büchi and de Mayo reactions, has found use in a plethora of total syntheses.^28–32^ Today, the [2 + 2]-cycloaddition is frequently used in synthetic efforts toward cyclobutane-containing target molecules and for compounds in which the carbon backbone can be constructed *via* a subsequent fragmentation/rearrangement of the cyclobutane ring. While the earliest examples of this reaction were realized by direct irradiation, energy-transfer (EnT) driven processes have also been developed as a part of advancements made with photoredox catalysts.^33^ Further developments include selective activation of Lewis-acid-bound substrates and control over relative and absolute stereochemistries.^34–38^ The generally accepted mechanism of the [2 + 2] cycloaddition commences with photoexcitation of one of the alkenes (either by direct irradiation or energy transfer photocatalysis), forming a biradical that then attacks the other alkene. While the combination of the two reagents could be a concerted cycloaddition, a stepwise mechanism, where the attack of a less-stabilized radical occurs first, has been supported by computational studies and the typical *anti*-relationship of the substituents.^39,40^

**Scheme 1:**
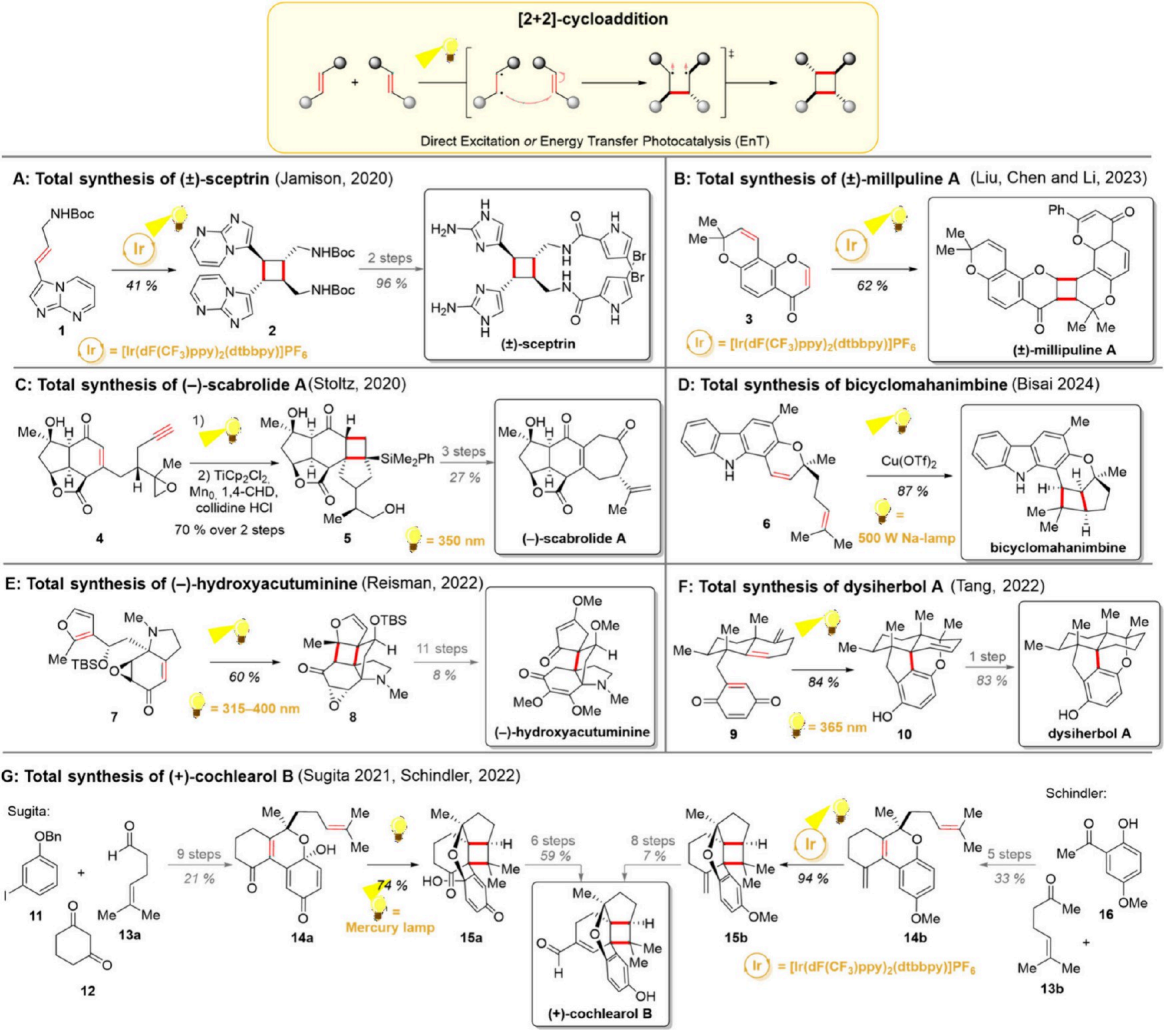
[2 + 2]-Cycloaddition (Upper Part) and Its Utilization in Recent Total Syntheses

In natural product synthesis, the [2 + 2]-cycloaddition has remained relevant throughout the decades. In an intermolecular way, it represents an important fragment-coupling strategy, which is particularly used to achieve C_2_-symmetric natural products such as (±)-sceptrin (Scheme 1A) or other dimeric natural products belonging to families such as truxinates or lignans.^41,42^ Furthermore, homodimerization products can also be obtained by combining identical fragments via two chemically different alkenes (Scheme 1B).^43^ In an intramolecular manner, the [2 + 2]-cycloaddition often closes two new rings, a four-membered one and another whose length is decided by the connecting carbon chain. To this end, representative recent examples in natural product chemistry can be found from the total syntheses of (−)-scabrolide A, bicyclomahanimbine, (−)-hydroxyacutuminine and dysiherbol A (Scheme 1C–1F).^44–47^ Besides these examples, the [2 + 2]-cycloaddition has been used toward even more complex natural products, such as Taiwaniadducts, epolones, and synthetic efforts toward dodecahedrane.^48–51^

The question of whether the cycloadditions are realized with direct irradiation or *via* energy transfer photocatalysis warrants its own discussion. While direct irradiation approaches may be appealing when the substrate itself is the best light-harvesting species in the reaction mixture, the addition of a photocatalyst can be helpful, especially when the targeted functional groups would only weakly absorb. The importance of the slight structural modifications on the substrate were demonstrated in the two photochemistry-driven total syntheses of (+)-cochlearol B (Scheme 1G)^52,53^ From a strategic point of view, the groups of Sugita and Schindler both decided to use the [2 + 2]-cycloaddition of the tricyclic precursors **14a** and **14b** to create the cyclobutane ring present in the natural product. The synthesis of Sugita started with the iodobenzene **11** and cyclohexanedione **12**, giving the tricyclic diketone **14a** as a precursor for the cycloaddition in 9 steps.^52^ On the other hand, the optimized route of Schindler began with the acetophenone **16** and sulcatone (**13b**), yielding their tricyclic compound **14b** in 5 steps.^53^ Upon the key [2 + 2]-cycloaddition, only dienone **14a** would cyclize under direct irradiation, whereas the cyclization of chromene **14b** did not proceed in the absence of a photocatalyst. Based on the structure–reactivity studies by Sugita, it was rationalized that the presence of the electron-deficient para-quinone was crucial for the direct irradiation approach, perhaps due to its well-established light-harvesting properties. Eventually, the natural product was obtained in 15 steps (Sugita) or 14 steps (Schindler) as reported for the longest linear sequence. Importantly, the three-dimensionally rich nature of this natural product, together with the efficiency of the key [2 + 2]-cycloaddition reaction, has continued to inspire chemists, leading to another synthetic study by Zhao’s group in 2024.^54^

In some cases, the [2 + 2]-cycloaddition reaction can also be directly followed by further reactions, especially rearrangements, resulting in multistep reaction sequences. A driving force behind these transformations is often the strain-release from the cyclobutane ring combined with favorable absorption/redox properties of the cyclobutane intermediates. As a representative example, the Yang group reported in 2021 a photocatalyzed synthesis of cyclohepta­[*b*]­indoles via [2 + 2]-cycloaddition/retro-Mannich-type reaction.^55^ While previous work had established the successful dearomative [2 + 2]-cycloaddition of a derivative of **17** with energy transfer catalysis, strategic modification of the substrate was found to enable the follow-up ring opening.^56,57^ As the obtained products closely resembled the backbone found in various *Aspidosperma* alkaloids, the reaction was soon applied to the total synthesis of (±)-aspidospermidine (Scheme 2A).^58^ Toward this end, the spiropentene-containing indole **17** was identified as a suitable precursor for the [2 + 2]-cycloaddition/retro-Mannich reaction, which was initiated by one-electron oxidation of the substrate to **18**. This intermediate underwent a smooth [2 + 2]-cycloaddition to yield cyclobutane **19**, which was then reduced back to closed-shell amine **20**. A retro-Mannich reaction would then take place, resulting in expansion of the 4/4-fused ring system to a six-membered ring in **21**. The naturally occurring (±)-aspidospermidine could then be achieved in 8 steps.

**Scheme 2:**
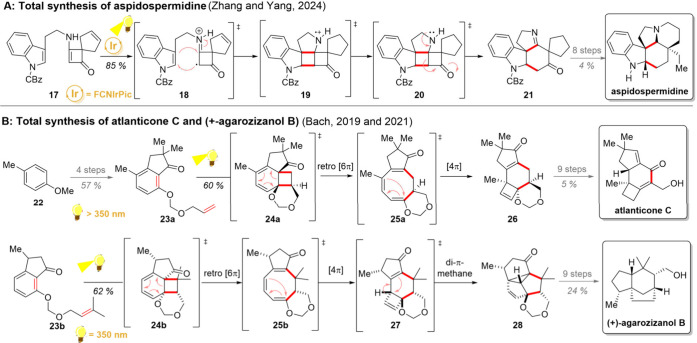
Use of [2 + 2]-Cycloaddition Initiated Rearrangements in Total Synthesis of Aspidospermidine (A), Atlanticone C (B), and (+)-Agarozizanol B (C)

Based on the preliminary report by Wagner,^59^ the Bach group reported in 2018 a photochemical sequence comprising an *ortho* [2 + 2]-cycloaddition, thermal disrotatory ring opening, and retro-[6π] cyclization.^60^ Importantly, this multiphoton cascade would give rise to a complex tricyclic hexahydrocyclobuta­[*i*]­chromene skeleton in a single step. The synthetic utility of this reaction was soon realized, and the following year this cyclization/rearrangement was utilized as a key step in the total synthesis of atlanticone C (Scheme 2B).^61^ Starting from *para*-cresol (**22**), the cyclization precursor **23a** was quickly assembled in 4 steps, which was then directly irradiated (λ ≥ 350 nm) to commence the desired photocascade. The *ortho* [2 + 2]-cycloaddition generated the cyclobutane **24a**, which then underwent a disrotatory ring opening to **25a** and [4π] cyclization to cyclobutene **26**. This photoproduct could then be converted to the desired natural product in 9 additional steps. Interestingly, upon further studies on this photocyclization/rearrangement, the Bach group later reported that changing the irradiation source to a shorter wavelength (λ = 350 nm) was able to initiate a further di-π-methane rearrangement following the afore-described photocascade.^62,63^ This cyclization reaction was also harnessed in natural product synthesis, this time toward sesquiterpene (+)-agarozizanol B.^64^ Thus, using a differently methylated intermediate **23b** underwent the usual *ortho* [2 + 2]-cycloaddition, retro [6π] cyclization, and [4π] cyclization to **27**. Under the short-wavelength irradiation, **27** further rearranged to pentacyclic **28**, which could be converted to (+)-agarozizanol B in 9 further steps or alternatively to jinkohol II in 14 steps.^65^ Notably, the photocyclization products **26** and **28** could also be utilized toward the enantioselective syntheses of the aforementioned natural products.^64,66^

### 2.2 Photoinitiated Diels–Alder Reactions

Diels–Alder reaction is a well-established thermal [4 + 2]-cycloaddition which has been extensively used in total synthesis for the generation of the carbon framework of natural products.^67^ However, unless particularly reactive starting materials with a clear electronically matching nature are used, the reaction requires heating to high temperatures and prolonged reaction times (often several days). As these forcing conditions present challenges with functional group tolerance and sensitive substrates, further developments have been made to address these issues. Herein, the photochemical methods can offer ways to activate one of the reaction partners or to generate one or another reaction partner *in situ*. For example, photocatalytic oxidation of an alkene to its radical cation can be used to accelerate or even enable the cyclization between two electron-rich partners.^68^ Photoenolization (Scheme 3, upper left) can be used to generate the dienol from benzaldehydes and benzophenones via excitation of the carbonyl group followed by intramolecular 1,5-HAT.^69–71^ On another note, by substituting one of the alkenes in diene with an alkyne, photoirradiation can be used to trigger a photodehydro-Diels–Alder reaction (Scheme 3, upper right).^72–74^

**Scheme 3:**
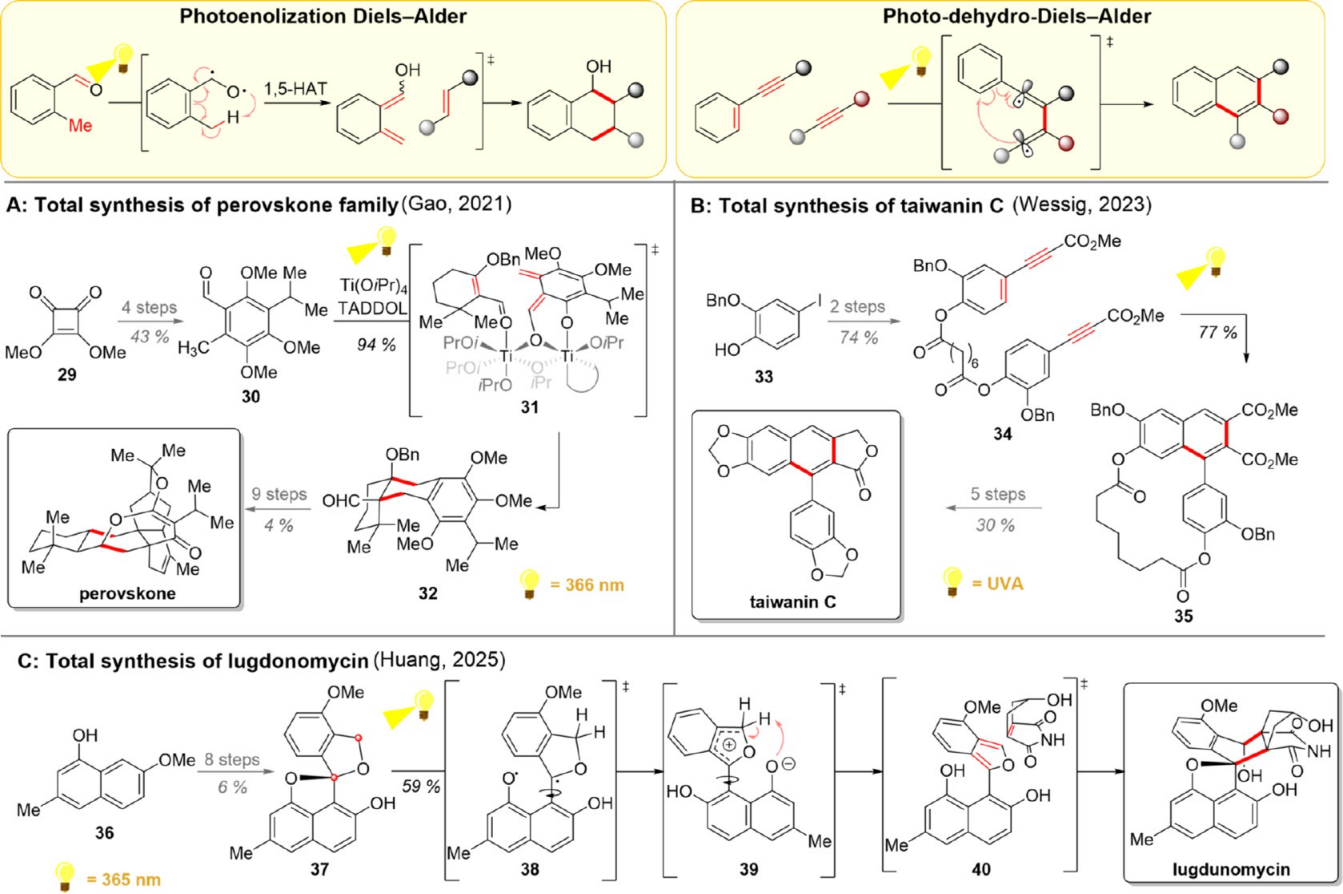
Photochemical Diels–Alder Reactions (Upper Part) and Their Uses in Total Syntheses of Perovskones (A), Taiwanin C (B), and Lugdonomycin (C)

Over the past years, the group of Gao has made significant improvements to the photoenolization Diels–Alder methodology. First, the titanium isopropoxide catalyst was introduced as a means of facilitating photoenolization and preorganizing the diene and dienophile before the reaction.^70^ Furthermore, the precoordination forged by titanium most likely acts to prevent the aldehyde photoenol dimerization, a challenge which is typically addressed with a notable excess of the aldehyde or its dropwise addition.^75^ A combination of the aforementioned titanium catalyst with an achiral TADDOL-derived ligand also gave rise to an asymmetric version of the photoenolization Diels–Alder reaction.^76^ This asymmetric induction was further employed in the collective total synthesis of perovskones and structurally related hydrangenones in 2021 (Scheme 3A).^77^ The longest linear sequence started with cyclobutene **29**, which was converted into the aldehyde **30** in 4 steps. For the key photochemical transformation, titanium-bridged asymmetric complex **31** was formed by simply combining the components, followed by photoenolization. The desired Diels–Alder reaction proceeded rapidly, yielding **32** in 94% yield and an impressive 97.7:2.3 enantiomeric ratio (er) after recrystallization. The tricyclic aldehyde **32** served as a common precursor for the six terpenoids, with the parent compound perovskone shown as a representative example.

The group of Wessig, in turn, has extensively studied the photodehydro-Diels–Alder (PDDA) reaction with their focus particularly on the synthesis of arylnapthalene lignans.^72–74^ In 2023, these research efforts led to the total synthesis of taiwanin C among three other structurally similar natural products (Scheme 3B).^78^ Upon development of the key PDDA reaction, the group observed that while the intermolecular reaction was inefficient, connecting the two reaction partners with a suberic acid tether would enable an efficient intramolecular reaction. Hence, the group commenced the total synthesis of taiwanin C with phenol **33**, which was dimerized and alkynylated to intermediate **34** in 2 steps. Based on their previous experience with photodehydro-Diels–Alder transformations, the group decided to run the conversion of **34** to **35** in a continuous flow setup. The desired product **35** was obtained in 77% yield as a single regioisomer and was converted to the desired natural product in 5 additional steps.

In 2025, Huang’s group reported an alternative photochemical method to initiate a Diels–Alder reaction as part of their synthetic studies toward lugdonomycin (Scheme 3C).^79^ Starting with naphthalenol **36**, the substrate for the photoinitiated Diels–Alder reaction **37** was prepared over 8 steps. The spiroketal **37** was then subjected to direct irradiation, which homolyzed the phenoxy C–O bond to give **38**, followed by an intermolecular electron transfer to give zwitterionic intermediate **39**. Subsequent proton exchange likely gave isobenzofuran **40**, and the Diels–Alder reaction with *iso*-maleimycin formed the lugdomycin after regeneration of the previously broken napthol C–O bond in silica gel. The calculated reaction pathways, intermediates, and transition states supported the afore-described mechanism. Furthermore, DMSO, a widely successful solvent in photochemistry, was necessary for the progress of the reaction. The authors hypothesized that the key factor here would be the hydrogen-bonding ability of DMSO, which stabilizes and prolongs the lifetime of isobenzofuran intermediate **40**. In contrast, the isobenzofuran intermediate could not be generated with either heat or acidic conditions.

### 2.3 Isomerization and Electrocyclization of Alkenes

A photochemical *E*/*Z*-isomerization of alkenes represents another benchmark reaction in photochemistry. While rotation around a C=C bond is forbidden in the ground state, excitation of the alkene either with direct irradiation or energy transfer photocatalysis promotes an electron from the π_C=C_ bond to a π*_C=C_ bond, effectively generating a biradical.^80^ This biradical intermediate can freely rotate around what is now a C–C single bond, and upon returning to the ground state, the double bond is regenerated in either *cis* or *trans* geometry. However, the steric clash between the double bond substituents in *cis*-alkenes typically forces the system to twist out of plane, disrupting the conjugation and shifting the UV–vis absorption of the *cis*-alkenes to lower wavelengths. In the presence of a photocatalyst, disrupted conjugation in *cis*-alkenes often increases the triplet energy, making energy transfer from the photocatalyst less favorable. Since the *trans*-isomer reacts more efficiently in both cases, the cis-isomer can accumulate in the reaction mixture over time. This reaction paradigm thus offers an easy way to achieve contrathermodynamic chemistry.^81^ In other instances, positional isomerizations of alkenes can be possible by a mechanism akin to the photoenolization discussed previously (Section 2.2). Here, the movement of the alkene out of conjugation is one of the key strategies allowing the accumulation of the product.

In 2024, both Bisai and Pan and Liu groups utilized the *E*/*Z*-isomerization of alkenes as an important step in their natural product synthesis endeavors. In the case of the Bisai group, the *E*/*Z*-isomerization was necessary en route to (−)-codeine as the Wittig olefination of **41** with (bromomethyl)­triphenylphosphonium ylide gave merely a 4:1 ratio of the *cis*-isomer **43** to the *trans*-isomer **42** (Scheme 4A).^82^ However, irradiation of this mixture in the presence of an iridium photocatalyst for 10 h gave over 90% yield of the desired *cis*-isomer. From here on, a cascade double Heck coupling took place, giving **59** after some further modifications (see Scheme 5). For the synthesis of (−)-artapilosine A, the groups of Pan and Liu decided to utilize the photochemical *E*/*Z*-isomerization to initiate their key electrocyclic ring-closing reaction (Scheme 4B).^83^ Toward this goal, piperonylic acid (**44**) was converted to the ester **45**, which could be subjected to direct irradiation. In the presence of iodine, an efficient *E/Z*-isomerization took place, followed by an electrocyclic ring-closure, giving the tricyclic intermediate **46** in 54% yield. This common intermediate was then successfully converted to both isomers of artapilosine A and artapilosine B.

**Scheme 4:**
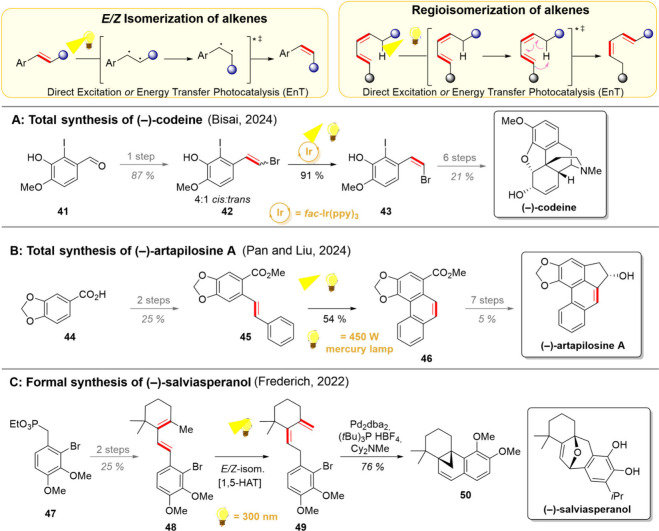
Alkene Isomerizations and Their Uses in Total Synthesis of (−)-Codeine (A), (−)-Artapilosine A (B), and (−)-Salviasperanol (C)

**Scheme 5:**
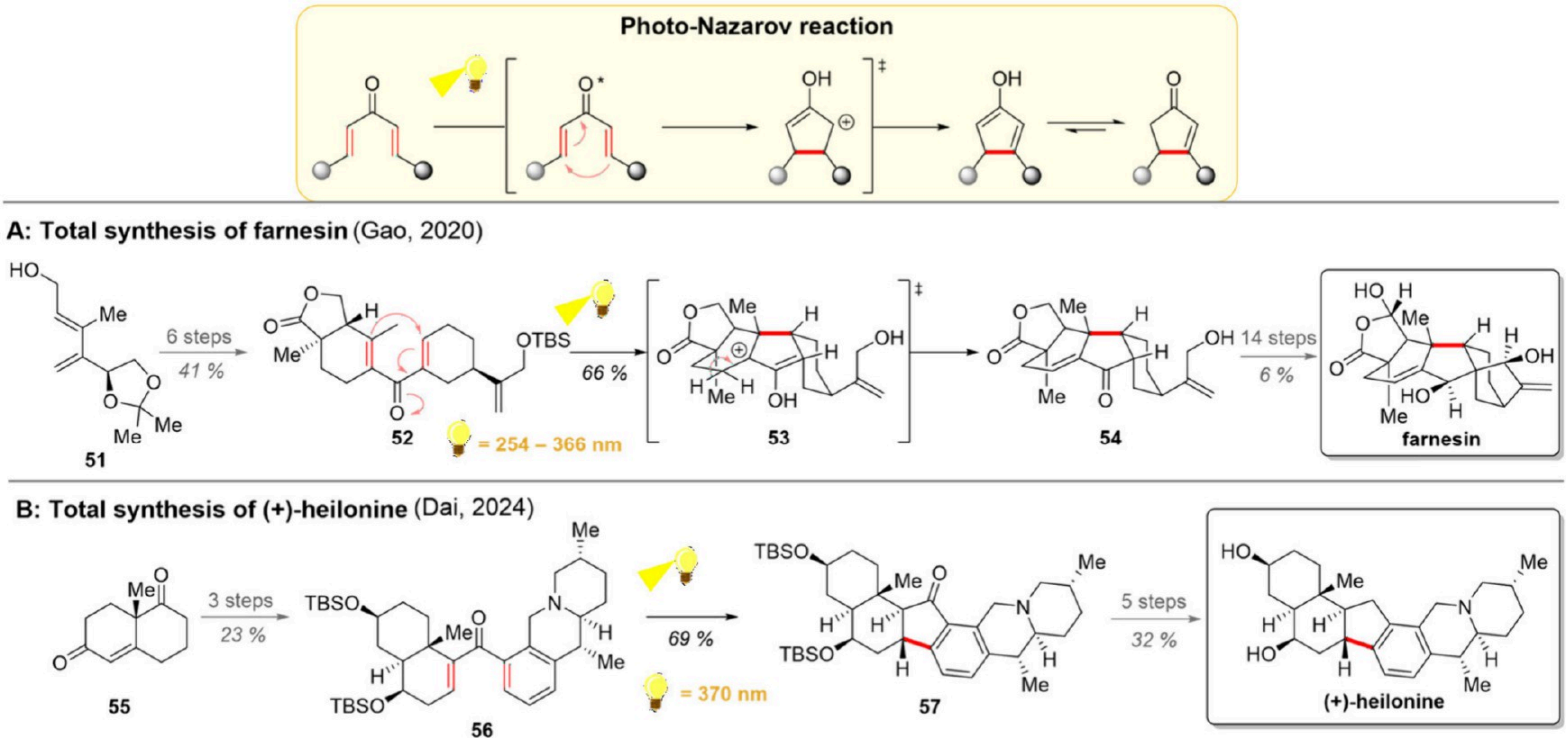
Photo-Nazarov Cyclization and Its Utilization in Total Syntheses of Farnesin (A) and (+)-Heilonine (B)

The strategy of positional alkene isomerization was harnessed by the group of Frederich in their formal synthesis of (−)-salviasperanol, a diterpene with a [6/6/6]-carbocyclic backbone (Scheme 4C).^84^ Inspired by Buchi’s earlier work on photoisomerization of β-ionone, the group opted to complement the previously established cationic and radical polyene cyclization strategies with a photochemical alternative.^85^ For this challenge, aryl phosphonate **47** was converted to β-ionyl derivative **48**. Upon exploring the key photoisomerization, the Frederich group noted that starting from either (*E*)-**48** or (*Z*)-**48** resulted in the same product **49**. Furthermore, during the reaction, (*E*)-**48** was depleted first, followed by full conversion of (*Z*)-**48** to **49**. These observations support the mechanism of the initial *E*/*Z*-isomerization of **48**, which is then followed by intramolecular 1,5-HAT. The authors also noted the metastable nature of **49**, which prompted the direct merger of a subsequent Heck bicyclization to give the tetracyclic **50** as the isolated product. This common intermediate could then be diverted to (±)-taxodione and to an intermediate previously used in the total synthesis of (±)-salviasperanol.

As a further example of an electrocyclization reaction of alkenes, recent advances with photo-Nazarov cyclization have attracted attention (Scheme 5, upper part). Over the decades, this [4π] electrocyclization has been identified as an efficient way for the cyclization of divinyl ketones to form synthetically useful cyclopentenones. The ground-state cyclization, which often requires activation of the carbonyl with Lewis acids, occurs in a conrotatory manner in accordance with the Woodward–Hoffman rules. In turn, the excited-state reaction occurs *via* disrotatory ring closure, allowing for the access to both cis- and trans-isomers from the same substrate by simply alternating between ground-state and excited-state processes.

Exactly this stereocontrol lies in the center of Gao’s total synthesis of farnesin (Scheme 5A).^86^ Drawing inspiration from the earlier synthesis of the Luo group, Gao and co-workers identified the synthetic promise of the Nararov-cyclization also to their target molecule.^87^ However, instead of the *anti*-ring junction described by Luo, to core to farnesin would require generation of the *syn*-isomer. Hence, the stereochemical outcome of the Nazarov cyclization was probed under both thermal and photochemical conditions, confirming the expected inversion in the relative stereochemistries of the reactions.^86^ The synthetic efforts toward farnesin were then initiated with a three-step generation of dienol **51**, which was further elaborated to the dienone **52** over 6 steps. Direct irradiation of this compound then resulted in the disrotatory cyclization to **53**, from which the tetracyclic framework of natural product **54** is obtained. The desired natural product could be achieved in 14 further steps.

The potential of photo-Nazarov cyclization was harnessed also a few years later, by the group of Dai in their total synthesis of (+)-heilonine (Scheme 5B).^88^ During their retrosynthetic planning, the group identified the photo-Nazarov cyclization as an efficient means to close the central 5-membered ring. With this strategy in mind, a carbonyl group was envisioned at the benzylic position, which could be removed by a late-stage reduction in the synthetic direction. The synthesis of the western fragment began with the Hajos–Parrish ketone **55**, which was converted into the dienone **56** over 3 steps. Applying the photo-Nazarov cyclization yields ring-closed hexacycle **57**. Subsequent steps then gave (+)-heilonine, making it the shortest total synthesis of the compound to date.

### 2.4 Oxidation of Alkenes

One-electron oxidation of alkenes gives rise to interesting intermediates possessing both ionic and radical character (Scheme 6, upper part).^89^ As electron-rich alkenes are the easiest to oxidize, the formation of electron-deficient radical cations can be seen as umpolung reactivity. Furthermore, while thermal alkene hydrofunctionalizations typically proceed to give Markovnikov selectivity, alkene radical cations offer easy access to anti-Markovnikov products. Hence, the reactions of alkene oxidation/nucleophilic coupling have been realized with a broad variety of nucleophiles and both intra- and intermolecularly.^90–93^ Although the one-electron reduction of alkenes has also been developed, it has found less utilization in recent total synthetic endeavors.^94,95^

**Scheme 6:**
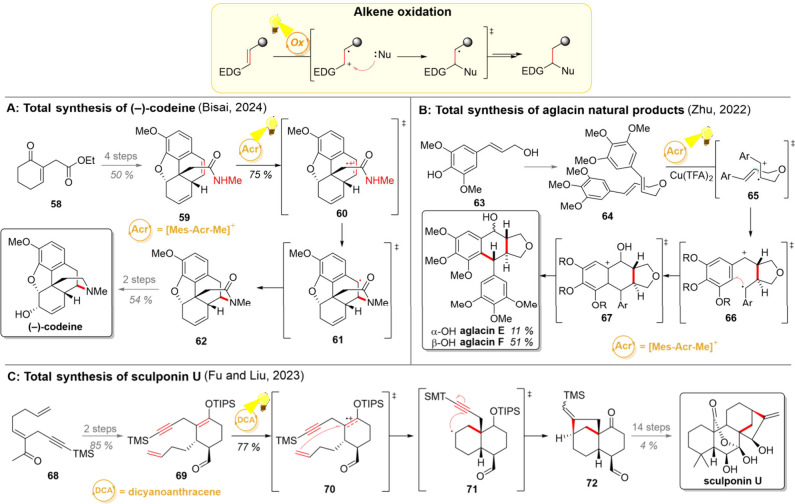
Photocatalytic Alkene Oxidation and Its Employment in Total Syntheses of (−)- Codeine (A), Aglacin Natural Products (B), and Sculponin U (C)

The total synthesis of (−)-codeine discussed in the previous section utilized another photochemical step to close the final ring system (Scheme 6A).^82^ From intermediate **59**, a single-electron oxidation of the benzylic double bond was used to form radical cation **60**, which was then intramolecularly trapped by the secondary amide. The benzylic radical in **61** was quenched with a thiophenol to produce intermediate **62**, which was two steps away from the (−)-codeine. Importantly, the efficiency of a highly similar synthetic strategy was utilized in the preparation of other morphinan alkaloids in 2023 by the groups of Banwell and White.^96^

Recently, the Zhu group demonstrated the use of one-electron oxidation of electron-rich styrene derivatives as a key strategy in their concise synthesis of aglacins (Scheme 6B).^97^ As widely distributed secondary metabolites, these dimeric compounds play diverse roles in plants, forming a defense against pathogens and pests as well as stimulating growth and development. In Nature, the biosynthesis of such products centers on regio-, diastereo-, and enantioselective radical cyclization; hence, a related transformation was chosen as a blueprint for the laboratory synthesis. Using sinapyl alcohol (**63**) as a starting material, its methylated dimer **64** was quickly assembled for the photocatalytic key step. The excited-state acridinium catalyst thus oxidized the alkene to form intermediate **65**, setting the reaction cascade in motion. A fast 5-*exo*-trig cyclization forged the first new C–C bond, followed by a 6-*endo*-trig cyclization on the aryl ring (proposed transition state **66**). During this cyclization sequence, a molecule of water attacks the benzylic carbocation, giving the hydroxy group present in the product. The aryl radical is eventually oxidized by the copper­(II) salt to give the delocalized aryl cation **67**, after which the aromaticity can be restored by deprotonation. Aglacin F is obtained as the major product from this cyclization cascade (α–OH 51%) while its β–OH epimer aglacin E is produced as a minor product (11%).

As another prototypical example of electron-rich alkenes, the groups of Fu and Liu utilized silyl enol ethers as precursors for the alkene oxidation (Scheme 6C).^98^ Starting their synthetic sequence with the ketone **68**, the silyl enol ether **69** was prepared in 2 steps. Following the initial report of the Mattay group, the generation of a cationic radical **70** initiated a cyclization sequence, leading to concomitant 6-*endo*-trig and 5-*exo*-dig cyclizations.^99^ This key sequence could be initiated with a combination of dicyanoanthracene photocatalyst with a phenanthrene cocatalyst, and the tetracyclic aldehyde **72** was generated in 75% yield. Further 14 steps then gave the desired natural product sculponin U.

### 2.5 Decarboxylation and Decarbonylation

Decarboxylation, the loss of CO_2_ from (organic) starting materials, has been used for a long time for the generation of reactive intermediates. Despite having a functional handle to mark the reactive site, thermal decarboxylations often require heating to high temperatures (>100 °C),^100,101^ putting sensitive substrates at risk of undesired decomposition. In contrast, under photochemical conditions, decarboxylation can be realized at room temperature using only a base and a (photo)­catalyst.^102–107^ While both the thermal and photochemical processes typically begin with an ionic deprotonation of the carboxylic acid, the decarboxylative step itself is best described as a radical process in photocatalytic reactions (Scheme 7, upper left).^100,108^ Hence, the photocatalytic decarboxylation results in a carbon-centered radical, which can be easily trapped with radical acceptors.^103^ If desired, the carbon-centered radicals can be converted back to ionic intermediates by one-electron reduction with the photocatalyst or by one-electron oxidation with a sacrificial oxidant.

**Scheme 7:**
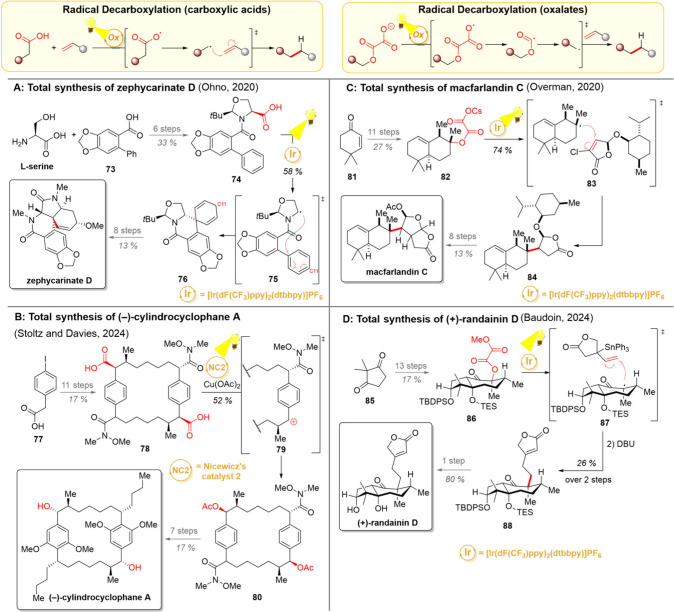
Photocatalytic Decarboxylation of Carboxylic Acids and Oxalates and Their Uses in Total Syntheses of Zephycarinate D (A), (−)-Cylindrocyclophane A (B), Macfarlandin C (C), and (+)-Randainin D (D)

A fine example of the use of photocatalytic radical decarboxylation in natural product chemistry was developed by the Ohno group during their total synthesis of zephycarinate alkaloids (Scheme 7A).^109^ l-serine and biphenyl carboxylic acid **73** were chosen as starting materials, giving carboxylic acid **74** in 6 steps. This carboxylic acid was then subjected to photocatalytic decarboxylation, resulting in the loss of CO_2_, followed by a 6-*endo*-trig radical cyclization (intermediate **75**). The secondary radical at C11 was then most likely briefly reduced to a carbanion, which underwent protonation to form spirocyclic compound **76**. Having achieved the reductive radical *ipso*-cyclization, zephycarinate D and its *N*-alkyl analogs could be easily accessed.

In 2024, the groups of Stoltz and Davies published their total synthesis of (−)-cylindrocyclophane A, a peculiar C_2_-symmetric macrocyclic natural product (Scheme 7B).^110^ While selective C–H functionalization strategies lie at the heart of this synthesis, clever photochemical “inversion” of carboxylic acids into acetates was also utilized. The synthesis began with benzylic acid **77**, which was converted into the macrocyclic compound relatively early in the synthesis (8 steps). After additional functional group adjustments, the bis-carboxylic acid **78** was accessed. Under photocatalytic conditions, decarboxylation was carried out, and the formed benzylic radical was oxidized to a carbocation **79** using copper­(II)­acetate as a terminal oxidant.^111^ Nucleophilic attack of the acetic acid cosolvent or of an acetate released at the reduction of copper­(II) then formed the acetamide **80**. The desired natural product was then achieved after seven additional steps.

Oxalates, 1,2-dicarboxylic acids, have also been found to be successful precursors for decarboxylation (Scheme 7, upper right). Synthetically, they represent another opportunity to utilize the oxidative decarboxylation reaction with substrates that do not initially possess a carboxylic acid group. Various alcohols, especially tertiary ones, have proven to be excellent precursors for oxalates. Their photochemical stepwise loss of two molecules of CO_2_ gives radicals on the initial *ipso*-carbon.^112^ Over the years, the group of Overman has harnessed the double decarboxylation of oxalates as a key method for fragment coupling in the total syntheses of (−)-solidagolactone and macfarlandin C (Scheme 7C).^113,114^ In conjunction with these syntheses, oxalates were established as the favorable redox-active tether, notably outperforming otherwise widely utilized phthalimide esters. The synthesis of macfarlandin C began with ketone **81**, which was converted to oxalate **82**. Regarding the choice of the redox-active tether, the sterically hindered nature of intermediate **82** was hypothesized to play a role in favouring oxalates over other alternatives. The double decarboxylation of the cesium oxalate generated a carbon-centered radical, which then attacked an electron-deficient alkene in a Giese-coupling manner (transition state **83**). Notably, the additional chloride substituent needed for smooth fragment coupling could be dehalogenated under the same reaction conditions. The targeted natural product was then achieved after 8 additional steps from **84**.

Decarboxylation of oxalates was also utilized in the 2024 total synthesis of (+)-randainin D by the group of Baudoin (Scheme 7D).^115^ Starting with dimethylcyclopentadione **85**, the oxalate **86** was prepared in 13 steps. Interestingly, the decarboxylative Giese coupling described by Overman proved unsuccessful here, giving the protodeoxygenated compound as the main product. The Baudoin group deduced that oxidative activation of the oxalate opened up the possibility for a single-electron reduction of the *alpha*-carbonyl radical as a way to close the iridium catalytic cycle and that the formed enolate would simply be protonated. On this note, the cesium oxalate was replaced by the methyl oxalate, which has been known to break heterolytically at a C–O bond upon one-electron reduction. This modification, together with the addition of a stannane group to the radical acceptor, led to a successful photocatalytic Pereyere–Keck type coupling (transition state **87**). While the light dependence of this transformation was established, the exact mechanism of the reaction remains to be clarified, since the homolysis of methyl oxalates can take place either photocatalytically or by tin radicals. After the successful fragment coupling, global deprotection of lactone **88** gave the desired natural product.

Aldehydes, ketones, and cyclic esters can also lose their carbonyl groups under light irradiation. While the photochemical strategies for aldehyde decarbonylation date back to the 1960s,^116–119^ recent advances in the methodology have enabled the use of a wider array of starting materials and photocatalysts to enhance this transformation.^120–122^ With aldehydes, the reaction often commences with a HAT from the aldehyde C–H bond, initiating the radical decarbonylation.^121,123^ With ketones, in turn, the a (partial) attack of an oxygen nucleophile, such as DMSO, to the carbonyl carbon can greatly facilitate the following concerted CO_2_ elimination and C–C bond rearrangement.^122^ In the absence of the activating DMSO nucleophile, however, the double C–C bond dissociation forms two benzylic radicals that can either directly couple back together or escape the solvent cage to form cross-coupled products. To circumvent the formation of cross-coupled products, the groups of Houk, Garg, and Garcia-Garibay opted to carry out their decarbonylative reaction in the solid state. As a manifestation of the success of their approach, the decarbonylation protocol was further utilized as a key step in the total synthesis of phychotriadine (Scheme 8).^124,125^ The synthetic efforts started with the malonic ester **89**, from which the ketone **90** was obtained in 5 linear steps. Interestingly, the groups found that the nature of the *N*-substituents had a significant impact on the photodecarbonylation process. With the *N*-Me groups, the desired C–C coupling product was obtained only in trace amounts, while most of the mass balance stemmed from disproportionation of the biradical intermediate. While the primary amides on both sides completely prevented the reaction, a successful decarbonylation was achieved with the half-deprotected ketone **90**. Comparison of the crystal structures of **90** and its fully deprotected version revealed a notable difference in the dihedral angle between the breaking C–C σ-bond and the nearest C–C bond of the π-system, implying better hyperconjugation on the half-deprotected **90**. The photoproduct **91** could then be converted to psychotriadine in 4 steps.

**Scheme 8:**
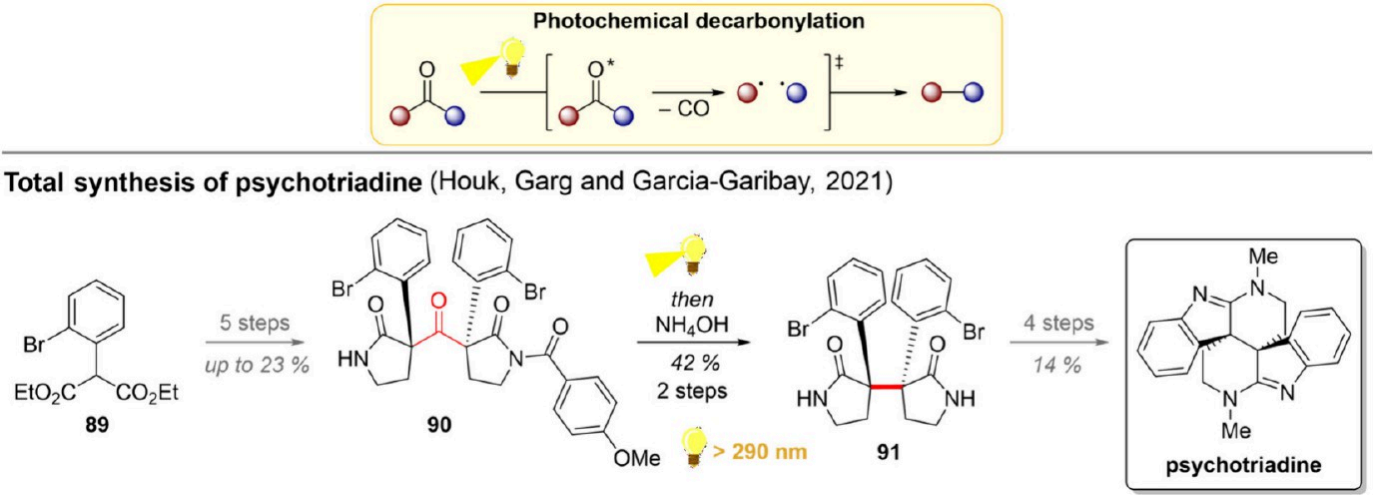
Photocatalytic Decarbonylation and Its Use in the Total Synthesis of Psychotriadine

### 2.6 Redox-Active Ester Fragmentation

The earliest accounts of the photoactivity of *N*-phthalimide esters were reported in the 1970s, when Kanaoka and co-workers extensively studied the light-induced reactions of their derivatives.^126^ While these initial studies mainly focused on transformations incorporating the phthalimide moiety to the product, the possibility for homolytic radical generation was soon demonstrated by the group of Skell with N–Br phthalimide esters.^127^ This finding in particular paved the way for the use of phthalimide esters for the generation of alkyl radicals and the broad variety of applications we see today.^128–131^ Although many methods for direct photocatalytic decarboxylation have been established, they typically begin with one-electron oxidation. With phthalimide esters, in contrast, the redox-moiety is first activated by one-electron reduction, followed by homolysis of the weak N–O bond and subsequent decarboxylation (Scheme 9, upper part). As the catalytic cycle commences with a reduction, the cycle-closing step becomes oxidative in nature. In this sense, phthalimide esters can be regarded as internal terminal oxidants that are typically highly compatible with a variety of organic substrates and reaction conditions. In addition to carboxylic acids, phthalimide esters of oxalates and alcohols can also be utilized, giving an alternative access to C- and O-centered radicals.^132^ So far, the phthalimide esters have also appeared to be the most favored redox-active tethers in total synthesis endeavors, although alternatives such as 4-substituted Hanzsch esters and *N*-alkylated pyridines (Katrinzky salts) have also been developed.^133,134^ This could be at least in part due to the ease and typically high yields obtained from their preparation, combined with well-studied properties and the variety of activation methods.^135^

**Scheme 9:**
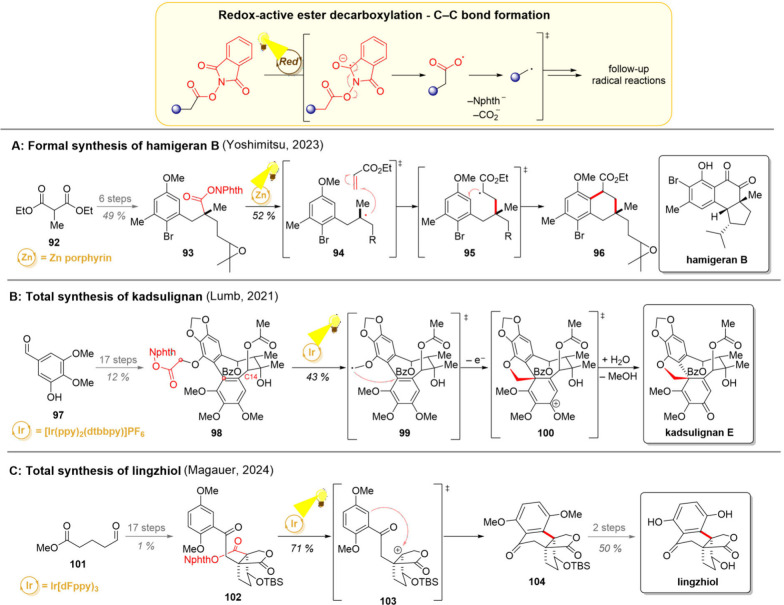
Photocatalytic Radical Generation by Phthalimide Ester Fragmentation and Its Application in Total Syntheses of Hamigeran B (A), Kadsulignan (B), and Lingzhiol (C)

A recent example for the use of phthalimide esters in the natural product synthesis can be found from the work of the Yoshimitsu group (Scheme 9A).^136^ In their efforts toward the formal synthesis of hamigeran B, phthalimide ester fragmentation was chosen as a method to generate a radical for the key Giese coupling/cyclization step. Starting from the malonate ester **92**, the intermediate **93** was formed in 6 steps (longest linear sequence). Photochemical degradation of the redox-active ester with a zinc porphyrin catalyst afforded the carbon-centered radical **94**, which was then trapped with ethyl acrylate to generate the radical intermediate **95**. A subsequent 6-*exo*-trig cyclization of the anisole derivative yielded the bicyclic product **96**. Notably, natural sunlight could also serve as the irradiation source in this reaction. The photoproduct **96** was then converted to an advanced intermediate used in a previous total synthesis of hamigeran B by the Miesch group, hence completing the formal synthesis.^137^

Phthalimide esters were further used as a precursor to generate α-oxygen radicals in a collective synthesis of various highly oxidized dibenzocyclooctadiene (DBCOD) natural products by the group of Lumb (Scheme 9B).^138^ Compounds bearing the DBCOD core are divided into many (sub)­families, yet this shared backbone has been postulated to account for their bioactivity prized in traditional Asian and Eastern European medicine. Furthermore, based on the biosynthetic hypothesis, addition of a nucleophilic methyl radical to an electron-rich aromatic ring (similar to that shown in **99**) is likely to play a crucial role in the synthesis of these compounds. As the cyclization from radical **99** onward is oxidative in nature, a reductive method for generating radicals would be necessary to achieve overall redox neutrality in a laboratory setting. Lumb’s collective synthesis toward these natural products commenced with the aldehyde **97**, which was converted to the phthalimide ester **98** over 17 steps. Gratifyingly, the reductive formation of the methyl radical led to an efficient 5-*exo*-trig cyclization (**99**). Oxidation of the so-formed aryl radical most likely took place (intermediate **100**), after which the regioselective addition of water followed by methanol elimination formed the desired *para*-spirodiene natural product. The strategic importance of this radical cyclization was further demonstrated by the same group, as modification of the substituent pattern on the C14 acetate in **98** led to the synthesis of six other DBCOD natural products.

A final example for the use of *N*-phthalimide esters in natural product chemistry comes from the total synthesis of lingzhiol and other *Ganoderma* meroterpenoids by the Magauer group (Scheme 9C).^139^ Starting from the aldehyde **101**, the phthalimide ester **102** was constructed in 17 steps, one of them comprising an intriguing photo-Fries rearrangement for the conversion of an intermediate aryl ester to a 2’-hydroxyacetophenone derivative. A second photochemical step was then utilized for a Friedel–Crafts type conversion of **102** to **104** following a procedure reported by Doyle.^140^ Herein, the reductive activation of the *N*-phthalimide esters was strategically harnessed to enable an oxidative second step. According to the proposed mechanism, the reductive activation of the phthalimide ester yields a carbon-centered radical, which is oxidized to the corresponding carbocation **103**. Intramolecular attack by the electron-rich aromatic ring then follows, forming advanced intermediate **104**. An alternative mechanism, where the tertiary radical attacks the aromatic ring (followed by oxidation to aryl cation and deprotonation) could also be possible, however, detailed mechanistic analysis was beyond the scope of the work. With tetracycle **104** in hand, lingzhiol was accessed after global deprotection.

### 2.7 Dehalogenation

Photochemical dehalogenation offers a mild and regioselective method for the generation of various alkyl and aryl radicals (Scheme 10, upper part). The reactivity of halogens toward this transformation decreases in order I < Br < Cl < F, making many iodides and bromides photolabile under low-wavelength irradiation.^141,142^ While the bromides and iodides often dehalogenate under direct irradiation or *via* a single-electron reduction with a broad variety of photocatalysts,^143,144^ dechlorinations and defluorinations typically require special reaction conditions, catalysts or substrates.^145–147^ In terms of the carbon skeleton, a benzyl ring, an α-carbonyl group, or the presence of *para*-EWG in a phenyl ring significantly facilitates the dehalogenation process.^148^ Out of these cases, the α-carbonyl one is particularly useful, as this position can be first halogenated by radical bromination or iodination, followed by dehalogenative radical generation.

**Scheme 10:**
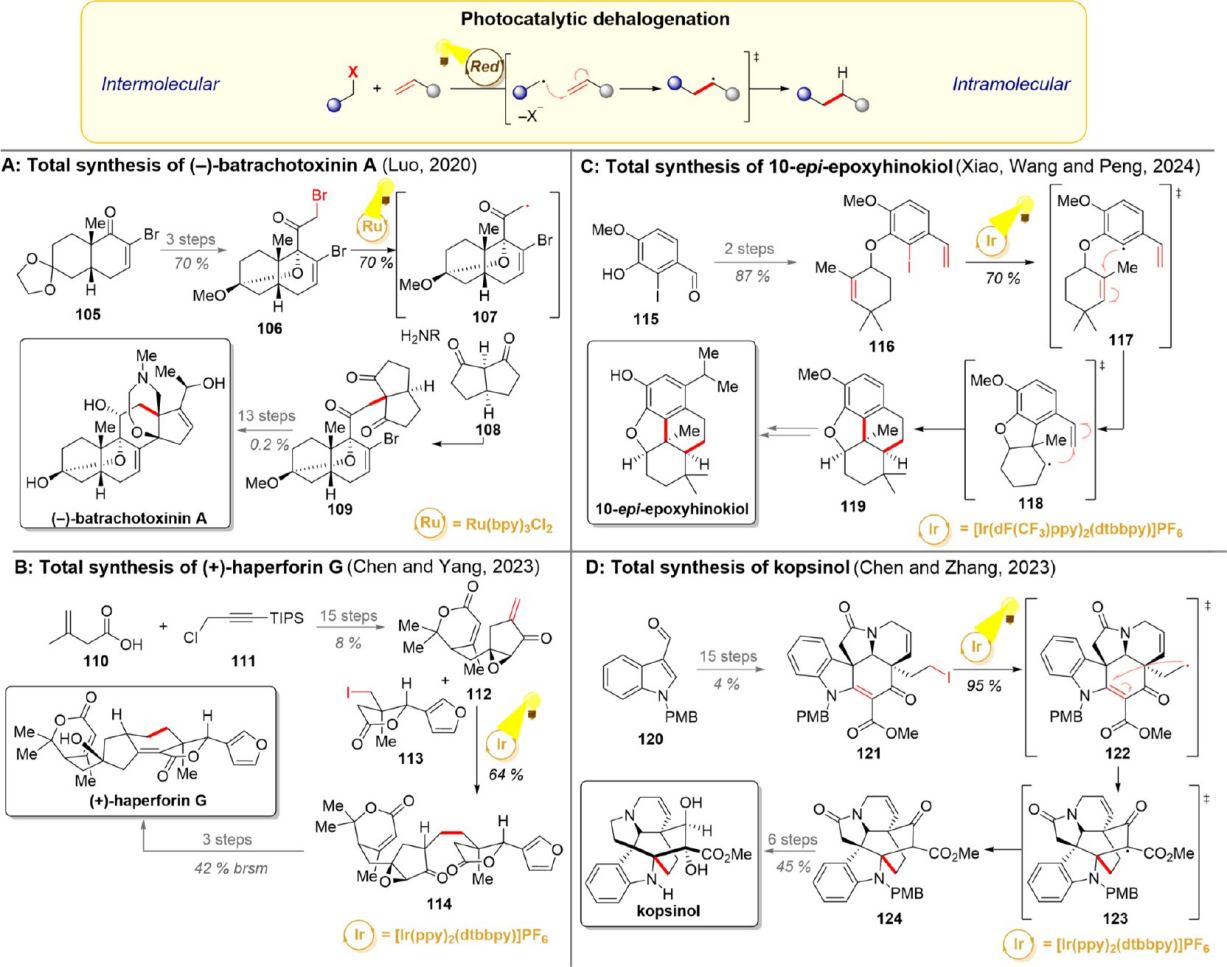
Photocatalytic Radical Dehalogenation and Its Application in Total Syntheses of (−)-Batrachotoxinin A (A), (+)-Haperforin G (B), 10-*epi*-Epoxyhinokiol (C), and Kopsinol (D)

In the realm of natural product synthesis, dehalogenative reactions can be utilized to initiate both inter- and intramolecular reactions. The intermolecular variants offer yet another strategy for fragment coupling; however, this time, the sequence-initiating step is reductive in nature. This strategy was exemplified in the total synthesis of (−)-batrachotoxinin A (Scheme 10A).^149^ Starting from a derivative of (+)-Hajos–Parrish ketone **105**, the group of Luo prepared the ketobromide fragment **106** in 3 synthetic steps. While their initial plan was to couple the bromide **106** with the diketone **108** in an S_N_2-reaction, attempts toward this transformation proved unsuccessful. However, the alternative photocatalytic strategy of reductive *alpha*-carbonyl radical generation followed by radical trapping with an enamine provided a 70% yield of the desired product **109** on a gram scale.^150,151^ Afterward, the natural product could be reached with 13 additional steps.

As another example, the groups of Chen and Yang utilized a similar dehalogenative Giese-coupling protocol for their total synthesis of (+)-haperforin G (Scheme 10B).^152^ The longest linear sequence in the enantioselective synthesis commenced with vinyl acetic acid **110** and chloride **111**, from which unsaturated ketone **112** was formed in 15 steps. This time, the photocatalyzed dehalogenation was carried out by a single electron transfer to alkyl iodide **113**, and the thus formed carbon-centered radical reacted further in a Giese-coupling manner. The coupled product **114** could be converted to (+)-haperforin G in 3 further steps.

Intramolecular dehalogenation-initiated reactions are often utilized in ring-closing reactions. For example, the groups of Xiao, Wang, and Peng employed the dehalogenation reaction as a starting point for their key ring-closing cascade en route to 10-*epi*-epoxyhinokiol (Scheme 10C).^153^ In their synthetic plan, iodide **116** was accessed in two steps from aldehyde **115**. A single electron reduction of the iodide **117** generated an aryl radical, which underwent first a 5-*exo*-trig cyclization to **118** followed by a 6-*endo*-trig cyclization. The formed benzylic radical would eventually be reduced to a carbanion and protonated to yield compound **119**. From this intermediate, the target compound could be reached in 3 steps.

Dehalogenation was also used in the total synthesis of kopsinol, a complex monoterpenoid indole alkaloid (Scheme 10D).^154^ To access the highly fused [2.2.2] octane ring system, the groups of Chen and Zhang devised a strategy in which the bridging carbons would be built by a radical Giese-coupling reaction. Hence, a 15-step sequence was used to convert aldehyde **120** to pentacyclic iodide **121**. Irradiation of this intermediate in the presence of an iridium photocatalyst led to a reductive cleavage of the C–I bond, giving radical **122**. An intramolecular Giese-coupling then took place as shown, closing the [2.2.2] octane ring system in **124** in a high yield. The regioselectivity of this reaction is also notable as the conjugated diene is attacked exclusively in the presence of a nonconjugated alkene. The diketone **124** could then be transformed into a variety of alkaloids in the kopsia family, including kopsinol, as shown in the scheme.

### 2.8 Hydrogen Atom Transfer (HAT)

Photocatalytic hydrogen atom transfer (HAT) is an efficient strategy to activate C–H bonds that have a low bond dissociation energy (BDE) or are even completely unactivated. Especially in the case of unactivated C–H bonds, high selectivities can still be achieved, particularly with intramolecular reactions, where the 1,5-HAT *via* a six-membered transition state often prevails (Scheme 11, upper part).^155,156^ Hence, HAT offers an important complementary strategy to many two-electron methods, where the C–H or X–H bond acidity is the reactivity-determining factor. As shown in Figure 2A, weakened C–H bonds are often found, for example, alpha to a heteroatom (particularly O or N), at highly substituted carbons, or at allylic/benzylic positions.^157,158^ As for the possible hydrogen atom abstracting species, oxygen-, nitrogen-, and sulfur-centered radicals typically abstract hydrogen atoms from positions with mediocrely strong BDEs, whereas the decatungstate anion and chlorine radical are well-established strong hydrogen atom abstractors. Importantly, various activation strategies have been developed for the mild generation of these HAT active species. For the decatungstate anion, direct irradiation converts the W=O bond into a HAT-active biradical.^157^ Ligand-to-metal charge transfer (LMCT) can be used to generate radicals (Cl, RO, RCO_2,_ etc.) by homolysis of the metal–ligand σ-bond.^159^ Proton-coupled electron transfer (PCET) methods combine a simultaneous deprotonation and oxidation of the heteroatom (N, O), while similar stepwise mechanisms can also be used to oxidize nitrogen and oxygen anions.^160–162^

**Scheme 11:**
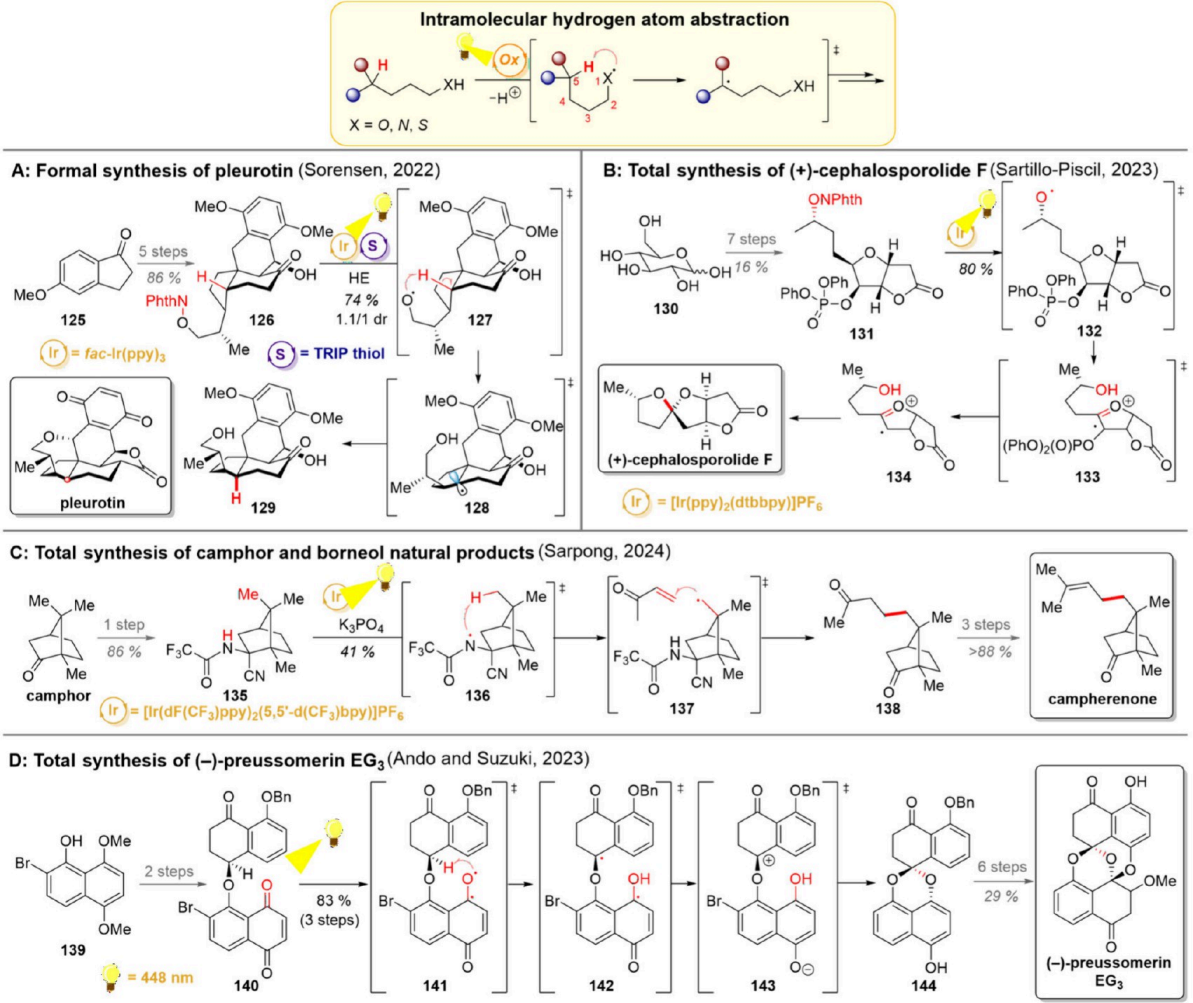
Intramolecular Hydrogen Atom Transfer and Total Syntheses of Pleurotin (A), (+)-Cephalosporolide F (B), Camphor Natural Products (C), and (−)-Preussomerin EG_3_ (D)

An example for the use of an intramolecular HAT in the realm of natural products can be drawn from the Sorensen group’s formal synthesis of pleurotin (Scheme 11A).^75^ The synthesis began with methoxyindanone **125**, which formed the southern part of **126** after coupling with the northern fragment. Importantly, the photoenolization/Diels–Alder reaction (Section 2.2) used for this fragment coupling inevitably produced the *cis*-fused hydroindane, whereas the *trans*-fused isomer would be needed for the natural product. Hence, an intramolecular 1,5-HAT was envisioned as a means for key epimerization. *N*-alkoxyphthalimide was chosen as the radical precursor, a decision influenced at least partly by the need for an OH-protecting group in the previous fragment-combining step. Now, reduction of **126** with a photoexcited iridium catalyst delivered short-lived alkoxy radical **127** as a hydrogen-atom abstracting species. While the regioselectivity of hydrogen atom abstraction could be well-regulated, control over the side for hydrogen atom back-donation proved to be more challenging (intermediate **128**). While the simple thiophenol mainly returned the *cis*-ring junction, sterically more hindered 2,4,6-triisopropylbenzenethiol could produce a higher ratio of the trans isomer (1.1:1 *trans*/*cis*). Gratifyingly, the diastereomers could be chromatographically separated, and two additional steps from **129** led to an intermediate previously converted to pleurotin by Hart and co-workers.^163^

A similar intramolecular HAT-strategy was further employed by the group of Sartillo-Piscil in the total synthesis (+)-cephalosporolide F (Scheme 11B).^164^ Their synthesis commenced with easily available starting materials d-glucose (**130**) and Meldrum’s acid, which were elaborated to the radical precursor **131** in a stereoselective manner. Similarly to the previous work of Sorensen, the *N*-phthalimide ester was reductively cleaved, giving an oxygen-centered radical **132**. An intramolecular 1,6-HAT to **133** took place, which results into a phosphate cleavage to give intermediate **134**. Finally, the radical was quenched by a HAT from a Hantzsch ester, and the hydroxy group performed an intramolecular spirocyclization to directly yield the natural product.

The Sarpong group, in turn, demonstrated in 2024 their use of intramolecular 1,5-HAT in the divergent total synthesis of camphor-derived natural products (Scheme 11C).^165^ In the heart of their synthetic strategy lies a selective functionalization of the bridgehead methyl group (marked in red) with the pseudoequatorial heteroatom. Despite the favored 1,5-relationship between the reaction-participating substituents, the overall strained nature of the [2.2.1]-bicyclic intermediate **136** could make the step less favorable, opening pathways for competing side-reactions. At least one of these, β-scission, could be hampered by choosing a nitrogen-centered radical over an oxygen-centered radical as the HAT active species, as the formed C=N double bond would be notably weaker than the C=O one. With these considerations in hand, the group began the optimization of the desired HAT step and the collective total synthesis. Hence, camphor was converted into the aminonitrile **135**, and after some optimization, deprotonation of this compound at elevated temperatures (60 °C) for 3 h was found crucial for obtaining high yields of the desired HAT-active radical **136**. This stepwise addition of reagents supports single-electron oxidation of the amide anion as the mechanism for the radical generation. Furthermore, the need for higher temperatures for the N–H deprotonation soon turned out to be an asset as the secondary C–H bond in the Giese-coupling product **138** would be weaker than the primary one in the starting material **135**, yet only minor amounts of double alkylation were observed. Further three steps furnished campherenone, a natural product that could be further converted to copacamphor.

The total synthesis of preussomerin EG_3_ represents an intriguing example where the substrate functions as both the light harvesting species and direct HAT active compound (Scheme 11D).^166^ The groups of Ando and Suzuki commenced their work toward this natural product with the bromonaphthol **139**, which was quickly elaborated to the napthaquinone **140**. Direct irradiation of this compound resulted in the excited-state naphthaquinone **141**, which rapidly underwent an intramolecular 1,6-HAT with the hydrogen atom near the quinone. An intramolecular charge transfer takes place to form zwitterion **143**, which cyclizes to give spirocyclic **144**. Notably, this HAT/cyclization process is highly stereoselective, indicating that intermediates **141**–**143** are too short-lived for rotation along the C–O bond to take place. After this successful photocyclization the target compound was accessed in 6 steps.

A powerful demonstration for an intermolecular HAT on strong C–H bonds came from the Wu group’s recent total synthesis of demissidine, a steroidal alkaloid isolated from several potato species (Scheme 12A).^167^ The commercially available tigogenin acetate (**145**) serves as an attractive starting point for the synthesis, having already the steroidal backbone in place; however, as a slight drawback, the stereochemistry of the C25 methyl group needs to be inverted. Both previously reported syntheses solved this problem by inserting a carbonyl group at C26, making the base-catalyzed epimerization possible. However, formation and removal of this carbonyl functionality notably lengthened the total syntheses.^168,169^ In contrast, the absence of carbonyl intermediates made the synthesis from Wu straightforward, and the amine **146** could be accessed in three steps from tigogenin acetate.^167^ For the key epimerization reaction, a strong HAT catalyst was required as the desired position at C25 was unactivated. The conditions reported by Wendlandt were chosen as a starting point.^170^ As expected, protonation of the tertiary amine proved necessary to deactivate the α-amino position C26, thus shifting the regioselectivity to C25.^171^ Gratifyingly, *in situ*-protonation of **146** with H_s_SO_4_ followed by the photocatalytic HAA/HAD-sequence afforded the desired axial-to-equatorial epimerization with 93% yield and diastereomeric ratio greater than 20:1. A simple hydrolysis of the southwest acetate group completed the total synthesis in just 5 steps and overall yield of 48%. Strategically, the epimerization strategy applied here demonstrates well the potential embedded in introducing HAT-driven epimerization as a complementary strategy to the acid/base approach.

**Scheme 12:**
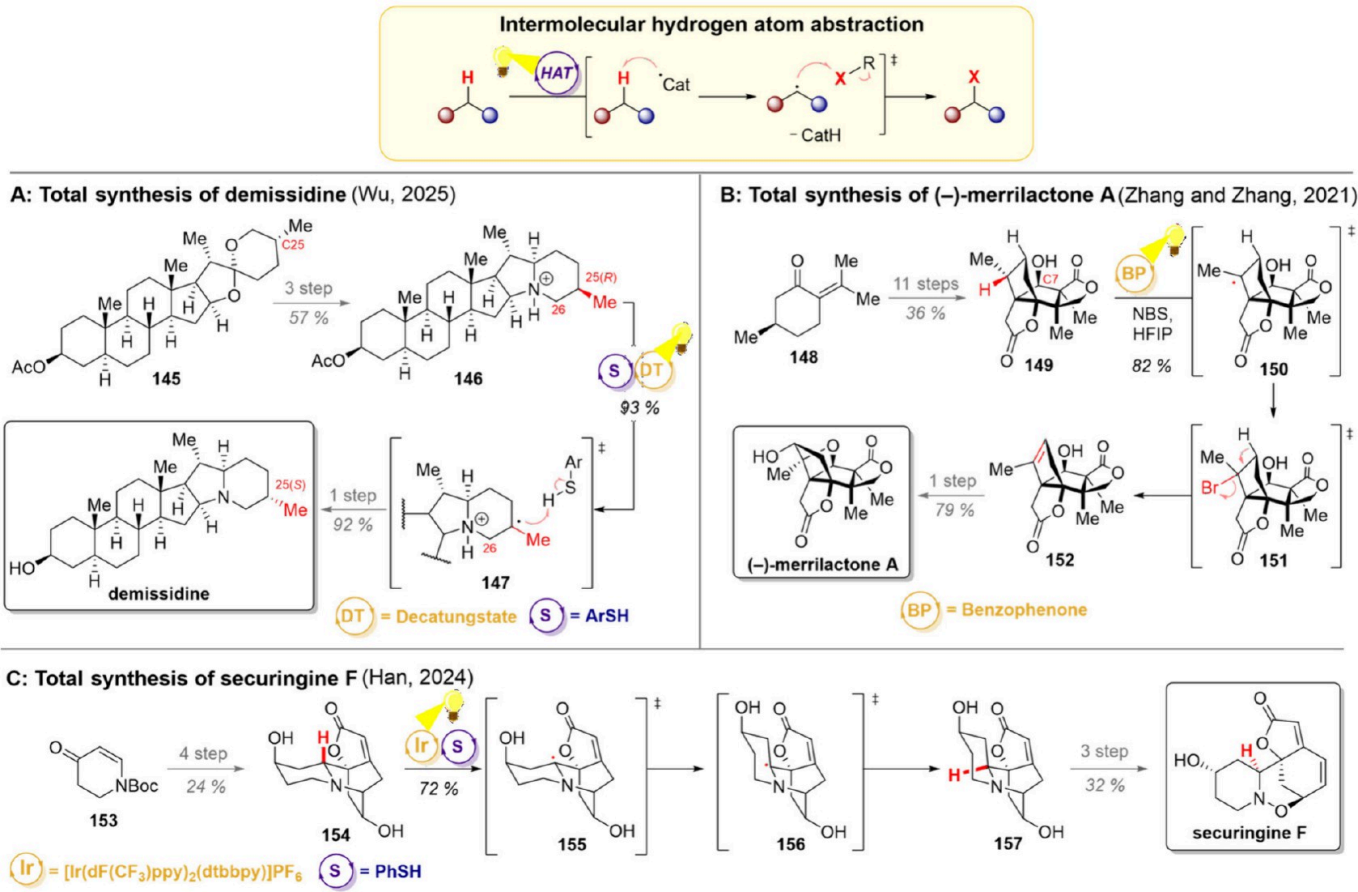
Intermolecular Hydrogen Atom Transfer and Total Syntheses of Demissidine (A), (−)-Merrilactone A (B), and Securingine F (C)

The groups of Zhang and Zhang demonstrated a somewhat unconventional application of HAT in their synthesis of (−)-merrilactone A, a sesquiterpene with promising results against neurodegenerative disorders (Scheme 12B).^172^ For the endgame of this synthesis, the groups decided to utilize a strategy where an unactivated alkyl motif would be desaturated, followed by redefinition of the stereocenter. To be successful, this transformation would need to overcome, particularly, the challenges in regioselectivity as no functional handles were planned to direct the desaturation. Using (*R*)-pulegone (**148**) as starting material, the tetracyclic lactone **149** was built in 11 steps, setting the stage for the pivotal desaturation. However, in addition to the desired C1–H bond, the compound **149** also possessed another weak *alpha*–OH C–H bond, thus presenting a possible challenge with regioselectivity. Indeed, initial attempts for the radical bromination using AIBN as radical initiator did not give any selectivity, but the desired C1 bromination was accompanied by oxidation of the C7-hydroxy group to the corresponding ketone. The groups then decided to harness solvent effects, more precisely the addition of hexafluoroisopropanol (HFIP) as a hydrogen bond donor, to reduce the electron density at C7 while creating a sterically hindering solvent shell around this position. Now irradiation of the reaction mixture in the presence of benzophenone as a photocatalyst and NBS as a brominating reagent gave desaturation product **152** in high yield. This synthetic intermediate proved particularly valuable as the late-stage modification of the oxygenation pattern could give rise to five natural products, among them the one-step conversion to (−)-merrilactone A.

As a final example, the group of Han utilized an intermolecular indirect HAT in 2024 in their total synthesis of securingine F (Scheme 12C).^173^ Their synthesis commenced with the Boc-protected enone **153**, which was converted to the diol **154** in 4 steps. For the key epimerization reaction, an iridium photocatalyst was employed to generate a thiyl radical, which abstracts a hydrogen atom from the relatively weak α-nitrogen C–H. The carbon-centered radical **155** then undergoes epimerization to **156**, after which the thiol back-donates the hydrogen atom to yield **157**. Taken together, this epimerization elegantly corrects the stereocenter set in an early fragment coupling (red hydrogen in **154**), thus allowing the total synthesis of securingine F to be completed in three further steps.

### 2.9 C–C Bond and C–Het Bond Fragmentation

In the synthetic planning, C–C bond fragmentation translates into a reconnection strategy during retrosynthetic analysis. Since the stereochemistry on cyclic substrates is often easier to control compared to their linear counterparts, controlling the stereochemistry in cyclic systems before moving to acyclic ones has proven to be a highly successful strategy in the synthesis of complex molecules. However, while the ring opening of cyclopropyl and cyclobutyl substrates is often driven by strain-release, rings such as cyclohexyls no longer benefit from this driving force, thus making their opening less favorable. Some of the well-established thermal methods for such fragmentations include the Grob fragmentation,^174^ ozonolysis,^175^ and various pericyclic processes including (but not limited to) *retro*-Diels–Alder, Claisen rearrangement with cyclopropane fragmentation and *retro*-ene reactions. Notable photochemical contributions to the field of cyclohexanol ring openings have been achieved with PCET and LMCT reactions.^176,177^ These methods also represent a wider reactivity pattern of alcohols: the initial generation of the oxygen-centered radical is typically followed by a β-scission, in which the formation of a strong C=O double bond makes up the energy loss of C–C σ-bond breaking (Scheme 13, upper part). The formed carbon-centered radical can then undergo trapping with radical acceptors or be quenched by a hydrogen-atom donor to yield the final product. As another intriguing entry, the group of Leonori recently developed a photochemical ozonolysis-resembling fragmentation of alkenes utilizing excited-state nitroarenes in place of ozone.^178^

**Scheme 13:**
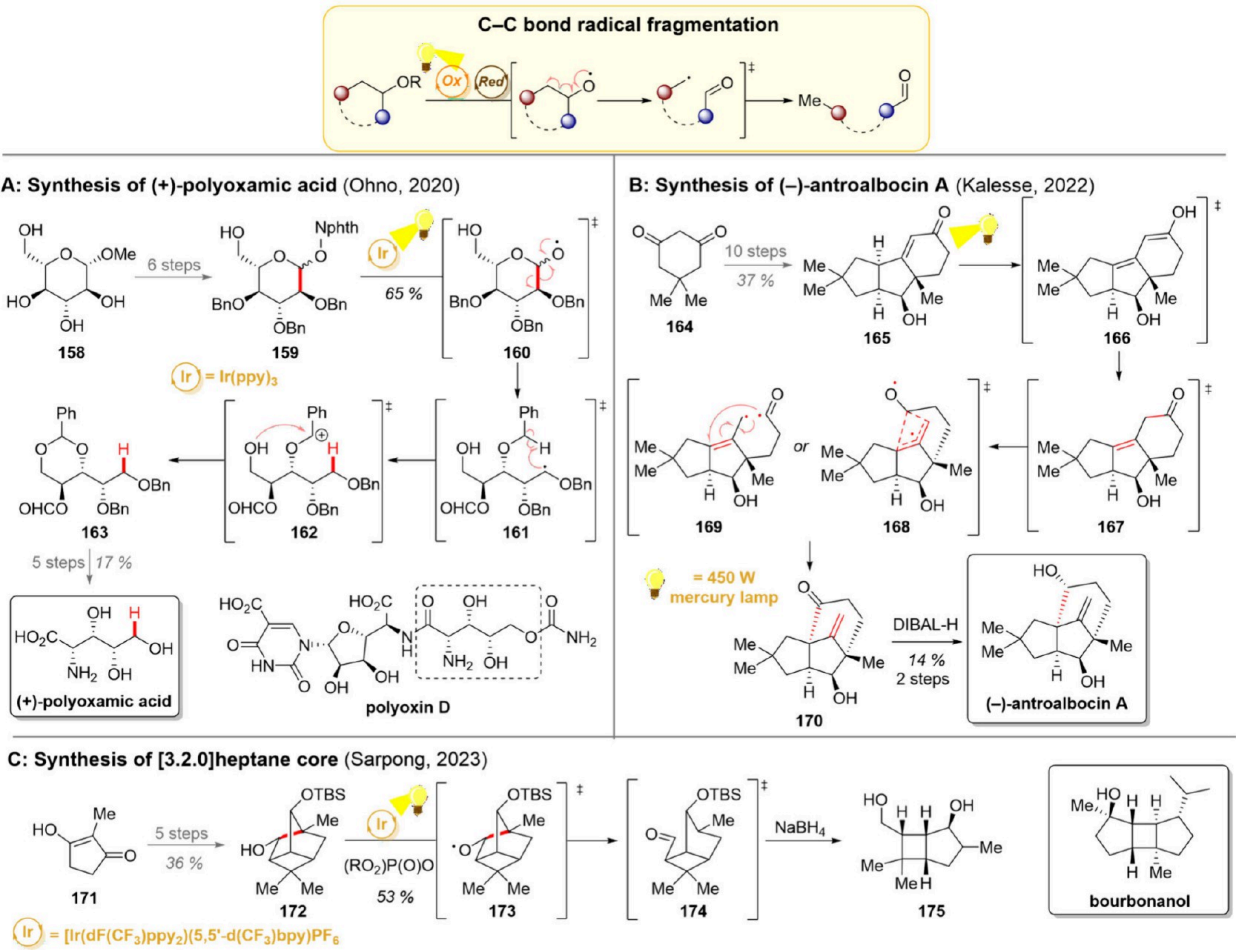
Radical C–C Bond Fragmentation and Its Utilization in Total Syntheses of (+)-Polyoxamic Acid (A), (−)-Antroalbocin A (B), and the Core of [3.2.0]-Heptane Skeleton Containing Natural Products (C)

The group of Ohno demonstrated the use of the ring-opening strategy in their synthesis of (+)-polyoxamic acid, a polyhydroxy α-amino acid (Scheme 13A).^179^ While this compound is not directly a natural product, it can be found embedded in numerous polyoxins, such as polyoxin D. Testing toward the feasibility of this synthesis started with methyl-α-d-glucopyranose **158** with all stereocenters in the synthetic target already in place. After stepwise insertions of suitable protecting groups, the pyranose was converted into the phthalimide ester **159** in 6 steps. For the photochemical step, a reductive activation of the phthalimide ester gave oxygen-centered radical **160**, which then underwent a ring-opening β-scission to **161**. A 1,5-HAT to the nearby oxygen protecting group then gave a benzylic radical, which was oxidized to benzylic carbocation **162** by the iridium catalyst. At this stage, nucleophilic attack from the primary hydroxy group concluded the reaction sequence, giving acetal **163**. Importantly, the presence of this unprotected alcohol was found crucial since in the absence of such a good nucleophile, the carbocation was attacked intermolecularly by adventitious water instead. Further substituent modification followed by global deprotection gave the (+)-polyoxamic acid.

A C–C bond breaking can also be employed as a first step in skeletal rearrangements. The group of Kalesse harnessed this strategy in their 2022 total synthesis of (−)-antroalbocin A, a novel antibacterial sesquiterpenoid isolated four years earlier (Scheme 13B).^180^ In line with the biosynthetic hypothesis, the bridged 5/5/6 tricyclic skeleton was envisioned to be constructed by a photochemical 1,3-acyl shift. Realization of the synthesis commenced with dimedone (**164**), which was converted to enone **165** in 10 steps. From here on, a direct irradiation of **165** was first proposed to form the enol tautomer **166** followed by protonation and migration of the alkene out of conjugation. The ketone **167** is then proposed to undergo the key 1,3-acyl shift, which, according to previous reports, can proceed either in a quasi-concerted (transition state **168**) or stepwise manner commencing with a Norrish Type I homolysis (transition state **169**).^181^ As the immediate photoproduct **170** proved highly unstable, the ketone was immediately reduced to give (−)-antroalbocin A in a 14% yield over both steps. Interestingly, further computational studies of the 1,3-acyl migration suggested an endergonic nature of the step, partly explaining the somewhat lower yields and instability of ketone **170**.

In 2023, the Sarpong group devised an interesting strategy of C–C cleavage to diversify highly strained tricyclooctanes (Scheme 13C).^182^ This 4/5 fused core can be found, for example, in the bourbon family of sesquiterpene natural products, such as bourbonanol.^183^ In the synthetic direction enone **171** was chosen as a starting material, and the tricyclo­[3.2.1.0^3,6^]­octane (**172**) was compiled in 5 steps including a [2 + 2] cycloaddition to form the four-membered ring. This alcohol was then subjected to a photocatalytic reaction with an iridium photocatalyst and a phosphate base present. The desired oxygen-centered radical **173** was formed either by a stepwise deprotonation-oxidation sequence or by a concerted proton-coupled electron transfer. A β-scission again occurred, opening the bridged ring system to bicyclic **174**. As the initially generated aldehyde in **174** was found to be highly unstable, it was reduced to the corresponding alcohol **175** before isolation.

Cleavage of a carbon-heteroatom bond can also be applied to numerous compounds as a means for generating reactive biradicals and initiating cascade reactions (Scheme 14, upper part). In 2022, the group of Nay realized the total synthesis of radulanin A, a chromene-resembling natural product with an expanded 6/7 fused ring system (Scheme 14A).^184^ With the relatively easy access to the phenolic part of the target compound in mind, the group started their synthetic efforts with benzaldehyde **176**. From here on, chromene **177** was achieved in 4 linear steps and with a good yield. Direct irradiation of the chromene resulted in a series of rearrangements, starting with the formation of quinone methide isomer **178**, which, upon further excitation, formed biradical **179**. An intramolecular 1,7-HAT to **180** followed, accompanied by an intramolecular 1,5-HAT giving **181**. From this intermediate, the final product could be achieved either directly or *via* cyclopropane **182**, which then undergoes a *retro*-Claisen rearrangement to give the desired natural product. Importantly, the approach of Nay represents a notable separation from the earlier syntheses where the 7-membered ring was closed by ring-closing metathesis or Mitsunobu reaction, highlighting the power of a rearrangement of the easily obtainable precursor.

**Scheme 14:**
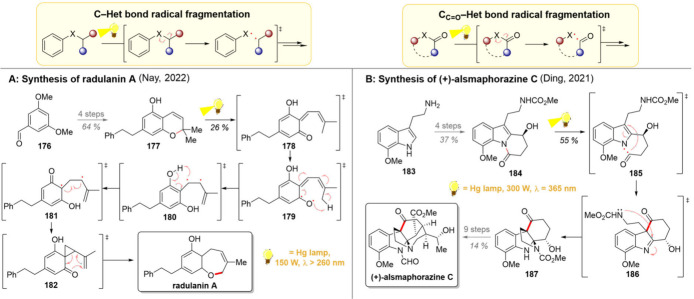
Radical C–Heteroatom Bond Fragmentation and Its Utilization in Total Syntheses of Radulanin A (A) and (+)-Alsmaphorazine C (B)

An amide C–N bond cleavage was harnessed by the Ding group in their total synthesis of (+)-alsmaphorazine C (Scheme 14B).^185^ Starting with methoxytryptamine **183**, a three-step sequence led to alcohol **184**, which contained all carbon atoms needed for the main core of the target molecule. Herein, direct irradiation of **184** resulted in the key photo-Fries rearrangement. C–N bond homolysis formed biradical **185**, which in turn underwent an intramolecular radical addition to the enamine group in tryptamine. Following this, imine **186** was then again intramolecularly captured by the linked carbamate, finishing the bridged tetracyclic ketone **187** as a main product together with its epimer. With the majority of the natural product’s cyclic framework now in place, the total synthesis could be concluded in 9 further steps.

### 2.10 Oxidation

Selective oxidation of unactivated C–H bonds represents a powerful tactic in the synthesis of target molecules, and particularly its expansion from enzymatic reactions to chemical laboratories equips synthetic chemists with highly valuable strategies for late-stage modifications.^186^ Since members of the same families of natural products often differ by their oxidation states and patterns, selective methods for oxidation can offer access to various natural products from a common precursor.^186–188^ As with thermal reactions, various chemical oxidants such as Cu^II^ salts (Scheme 7), peroxides, persulfates, or fluorinated organic compounds have proven compatible.^111^ Additionally, photochemistry offers facile means to convert triplet oxygen, which is abundant in room air, to its singlet state (Scheme 15, upper part). The thus generated reactive biradical can add to double bonds, forming metastable endoperoxides, which then often undergo disproportionation or rearrangements. Particularly from an experimental point of view, the use of molecular oxygen as a terminal oxidant represents significant ease of setup, cost-efficiency and accessibility.

**Scheme 15:**
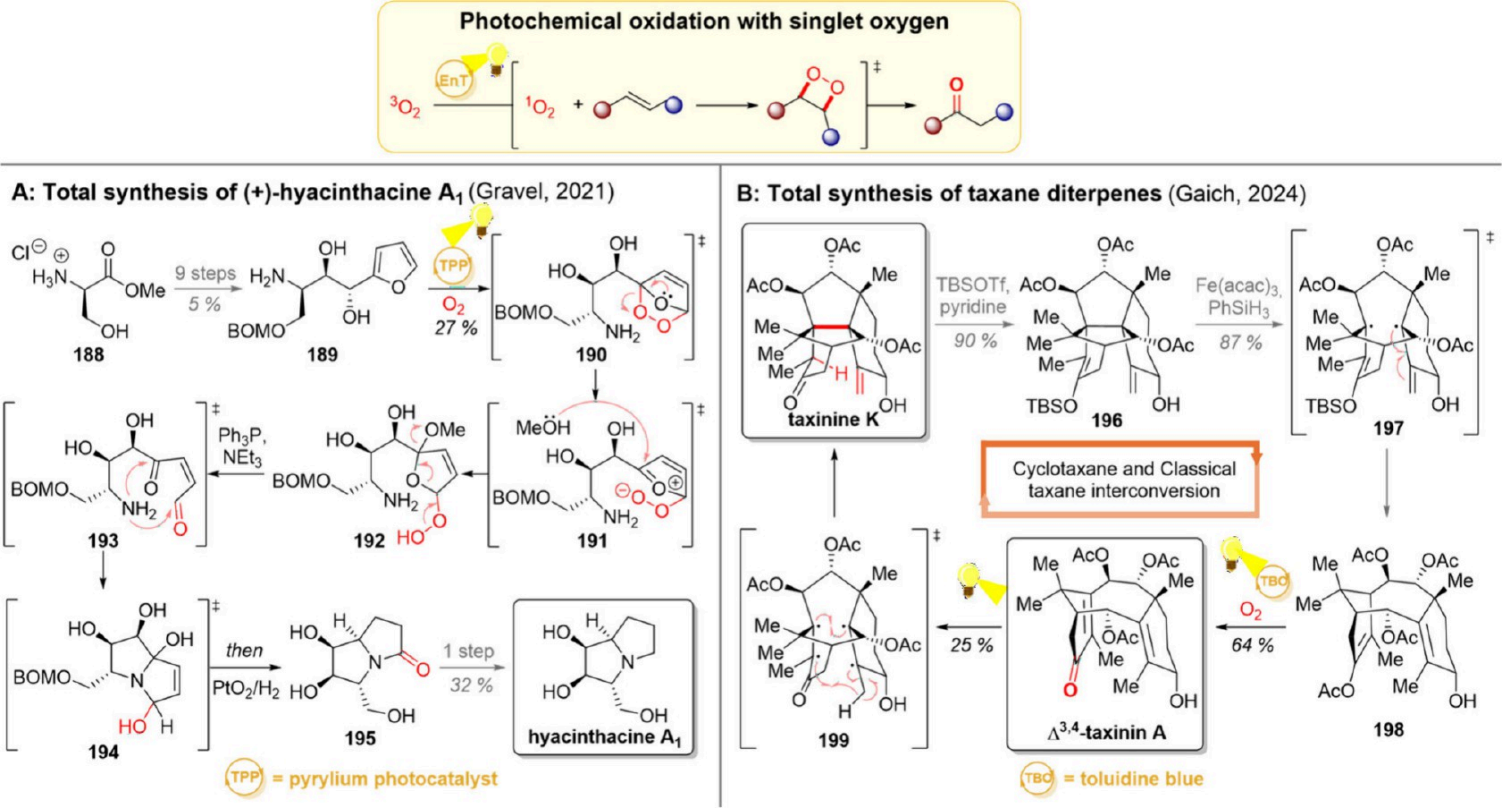
Photochemical Oxidation with Singlet Oxygen and Its Uses for the Total Synthesis of Hyacinthacine A_1_ (a) and in the Interconversion of Taxinine K and Δ^3,4^-Taxinin A (B)

The Gravel group demonstrated the utility of singlet oxygen in their 2021 total synthesis of (+)-hyacinthacine A_1_ (Scheme 15A).^189^ The group started their synthesis with serine methyl ester (**188**), and constructed the aminodiol **189** over 9 steps. The key photo-oxygenation then followed. Upon initial studies, the group determined that after the successful formation of endoperoxide **190**, a competing fragmentation could be initiated by the nearby hydroxy group. To circumvent this challenge, the reaction was carried out at low temperatures to achieve the desired hydroperoxide **192**. Due to the unstable nature of this photoproduct, an *in situ*-reduction to **193** was carried out, giving alcohol **194** after double ring-closure. Eventually, this intermediate was further reduced to **195**, which could be isolated in 27% yield over three steps. The desired natural product could be achieved from this intermediate with further reduction of the amide.

Sometimes oxidation reactions with singlet oxygen can be difficult to predict in advance. At the end of their impressive total synthesis of taxane natural products, the group of Gaich identified a synthetic loop to interconvert the cyclotaxane core to a classical taxane (Scheme 15B).^190^ The entry to this interconverting loop was obtained from taxinine K, which was synthesized over 26 linear steps. The interconversion sequence commenced by converting the taxinine K into its silyl enol ether **196**, possessing the parallel alignment of the π-bonds for the next reaction. Metal-hydride hydrogen atom transfer (MHAT) fragmented the central C–C σ-bond in **197** to yield **198**. Oxidation of this compound with singlet oxygen then finalized the conversion to Δ^3,4^-taxinin A. If needed, a further photochemical 1,5-HAT process could be used to return to taxinine K *via* intermediate **199**.

In addition to the use of singlet oxygen, the photochemical borylation-peroxidation sequence offers another method for the hydroxylation of organic starting materials. Gratifyingly, for photochemical endeavors, diboranes have proven to be efficient radical-capturing reagents. Hence, various photochemical methods can be utilized to generate the carbon-centered radical, which is then reliably trapped by the borylating reagent (Scheme 16, upper part).^191–194^ From a mechanistic perspective, diboranes can be further activated by coordination of, for example, fluoride anions^195^ or Lewis-basic solvent molecules.^196^ This photochemical radical trapping method offers a notable alternative to thermal borylations, which often utilize aryl halides or pseudohalides as starting materials in Miyaura-type reactions.^197^ While the following examples illustrate the potential of the photochemical borylation-oxidation in natural product synthesis, in principle, the borylated intermediates could be utilized for other downstream reactions, such as cross-couplings, as well.

**Scheme 16:**
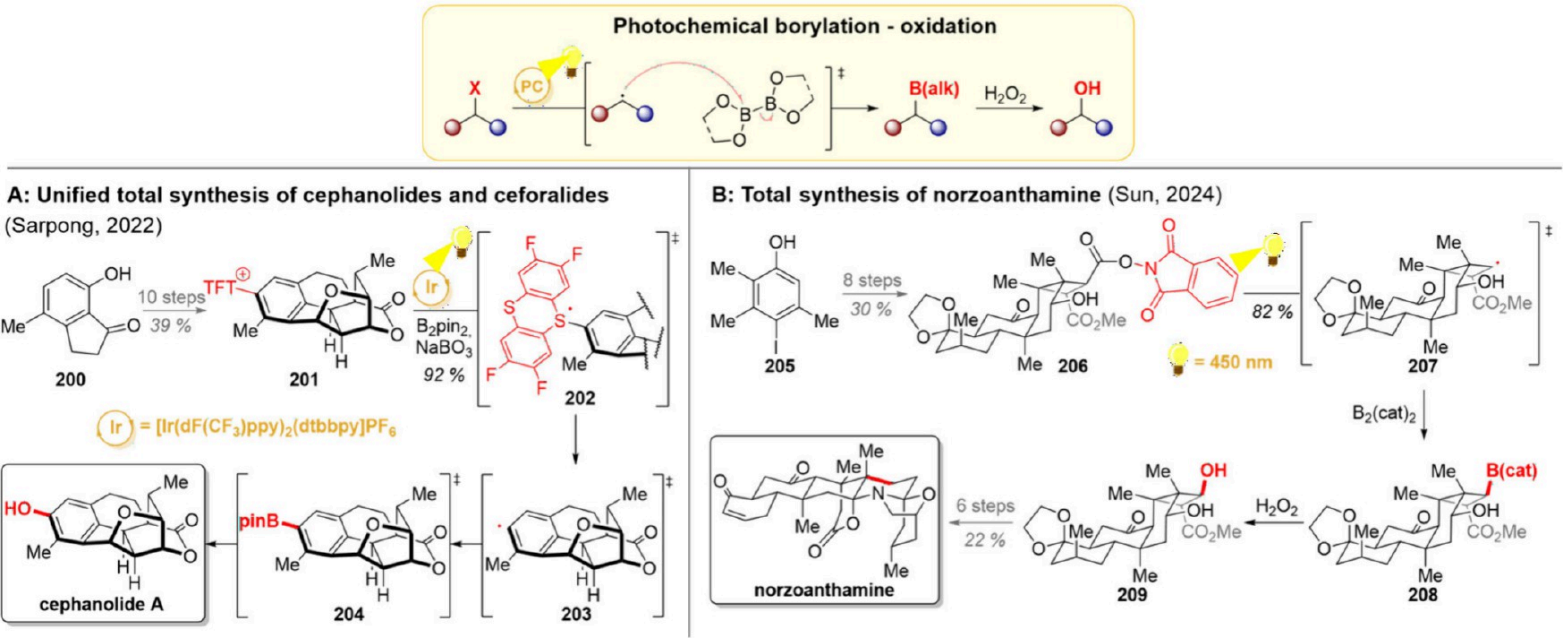
Photochemical Borylation-Oxidation Sequence and Its Utilization for Total Syntheses of Cephanolide A (A) and Norzoanthamine (B)

In 2022, the Sarpong group utilized the photocatalytic aryl borylation-oxidation sequence in their collective synthesis of cephanolides and ceforalides, benzenoid members of cephalotane-type norditerpenoids (Scheme 16A).^198^ Keeping Corey’s guidelines for retrosynthetic analysis in mind, the group decided to disconnect the maximally bridged ring first, leading to a significant simplification of the target compound. In the forward sense, this strategy proved extremely successful as the pentacyclic carbon framework of the natural product could be accessed in just 4 steps from hydroxyindanone **200**. Further modification, mainly on the bridged ring, furnished the cyclic ether **201** in 6 additional steps. One-electron oxidation of the thianthrenium salt **202** resulted in the degradation of the S–C_Ar_ bond to yield the phenyl radical **203**, which was trapped with borane to **204**.^198^ Subsequent oxidation to a phenolic hydroxy group gave the desired natural product in overall 11 steps. A particularly intriguing aspect of this last transformation is the generation of a highly unstable phenyl radical intermediate, a rather daunting task that has been greatly facilitated in the past decade.^192,199–201^

Another variant of the photochemical alkyl borylation was recently employed by the Sun group in their synthesis of norzoanthamine, a marine alkaloid with a broad range of biological activities (Scheme 16B).^202^ Their elegant synthesis featured altogether three photochemical steps: a dearomative-6π-desymmetrization early on in the synthesis, a [2 + 2] cycloaddition for the generation of the cyclobutane moiety, and photochemical borylation to make the handle for the construction of the cyclic amine. While the entire synthesis is certainly worth reading, we will here focus only on the last one of the photochemical steps. For this transformation, the phenol **205** was converted into the phthalimide ester **206** containing three of the rings found in the natural product. Direct excitation of the phthalimide ester led to homolysis of the weak N–O bond, and following decarboxylation gave radical intermediate **207**. This radical was then trapped by B_2_(cat)_2_ to give borylate **208**, and one-pot oxidation with hydrogen peroxide gave corresponding alcohol **209**. The natural product was then achieved in 6 additional steps, marking by far the shortest synthesis of norzoanthamine to date.

### 2.11 Metallaphotoredox Catalysis

Metallaphotoredox catalysis has become a prominent platform for combining the bond-forming capabilities of (transition) metal catalysts with light-driven reactions.^203^ In the realm of metallaphotoredox catalysis, the photocatalysts are often employed as a means to activate organic starting materials. Furthermore, a carefully planned SET between the photocatalyst and the metal catalyst can also be used to facilitate sluggish steps by either changing the oxidation state of the metal or promoting the whole complex to an excited state. Intriguingly, each metal strongly displays its own characteristics and is known to perform certain functions. In the following examples, the roles of copper, nickel, and cobalt in natural product synthesis are discussed in more detail.

Copper, as already alluded to a few times during this review, is well established to capture and oxidize transient carbon-centered radicals. In most cases, this is accomplished by adding copper­(II) salts to the reaction, which builds up a reservoir of persistent radicals ready to couple with the transient ones.^111^ After the radical capture, oxidation to corresponding carbocations follows, which are then easy to attack with nucleophiles. In 2023, the group of Zhu harnessed copper for the oxidation of a benzylic C–H bond during their total synthesis of (+)-stephadiamine, a morphine-resembling alkaloid with aza[4.4.3]­propellane core (Scheme 17A).^204^ Their total synthesis efforts commenced with napthol **210**, which was converted to tetracyclic *N*-Alloc derivative **211** over 12 steps. The oxidation of the benzylic position was then carried out from this intermediate following a protocol reported by the Yoon group.^205^ In this reaction, the iridium photocatalyst oxidizes the electron-rich aromatic ring to the corresponding aryl cation **213**. Loss of a proton shifts the radical to the benzylic position (**214**), after which it is oxidized to carbocation **215** by Cu­(II). At this point, the carbocation **215** was trapped by methanol in the original paper of Yoon, but the Zhu group reported that in their hands, the presence of adventitious water or oxygen resulted in the formation of ketone **212**. As the XRD analysis of the ketone **212** showed promising configuration in regards of the following steps, the reaction was optimized toward the ketone by adding 10 equiv of H_2_O as a nucleophile. With these modified conditions, the ketone **212** could be obtained in 83% yield and further converted to (+)-stephadiamine over 4 steps. As another example, the groups of Yang and Li opted to utilize phthalimide esters as their radical precursors while using copper­(II) as an oxidant toward their total synthesis of oxetanocin A.^206^

**Scheme 17:**
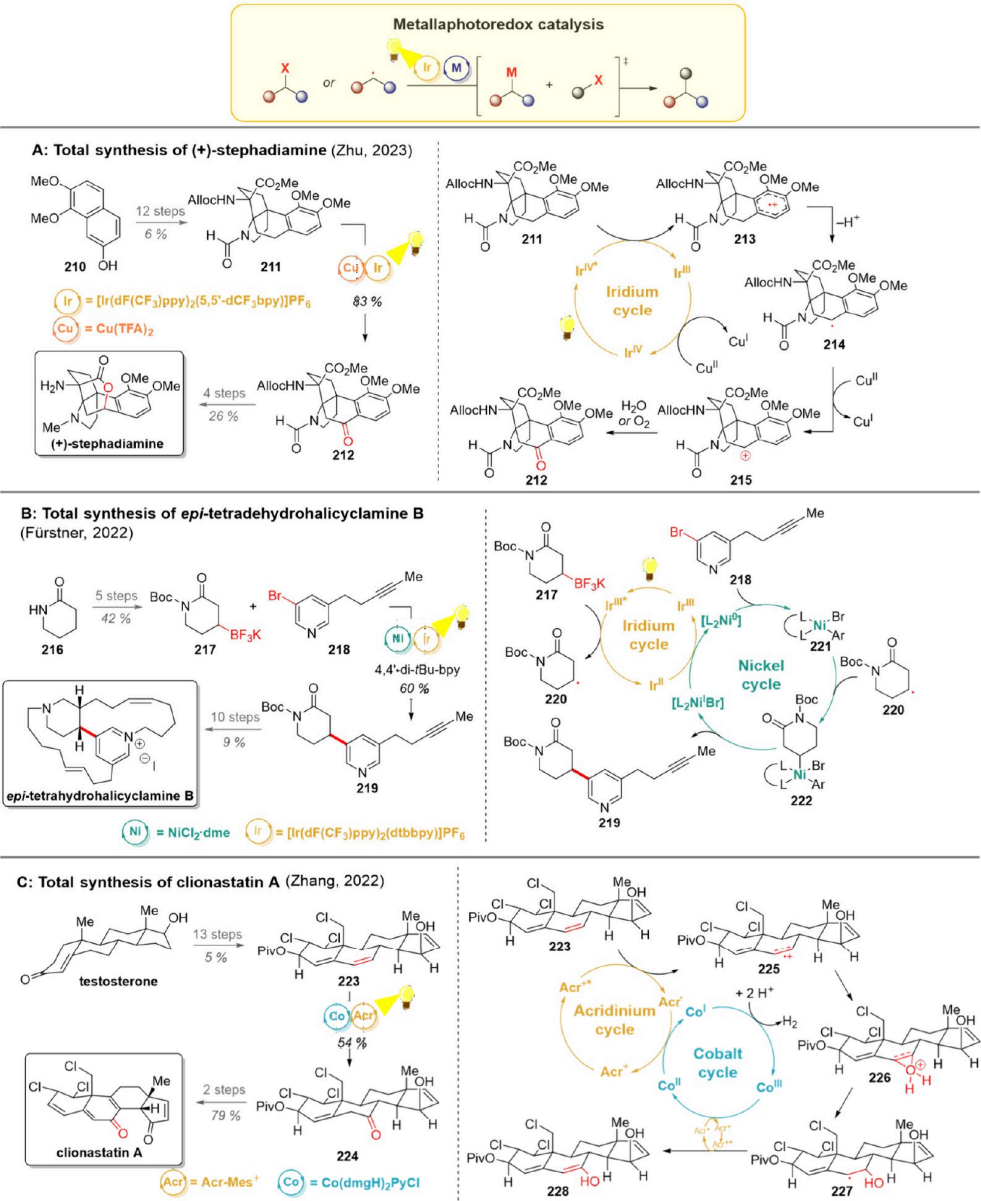
Examples of the Use of Metallaphotoredox Catalysis in Total Synthesis

Nickel photoredox catalysis has attracted significant attention over the past years, making it a versatile platform for numerous C–N, C–S, C–O, and C_sp2_–C_sp3_ coupling reactions.^207–209^ At the core of nickel photocatalysis is its ability to combine oxidative addition and reductive elimination steps to a cycle with efficient radical trapping. While some nickel photocatalysis can be achieved by employing nickel as both the light-harvesting and fragment coupling catalysts, the majority of the protocols rely on a combination of nickel and photocatalyst.^208,210,211^ In these dual catalytic approaches, the role of the photocatalyst (or LMCT catalyst) is typically either to generate the radical to be trapped by nickel or, in the case of nucleophilic additives, to activate or reactivate the nickel catalyst by energy transfer.^207,212–215^ The total synthesis of *epi*-tetrahydrohalicyclamine B by the group of Fürstner represents one of the examples of harnessing nickel photocatalysis in natural product chemistry (Scheme 17B).^216^ Despite extensive interest in the biosynthetic genesis, this exotic marine alkaloid had yet to be synthetically prepared. Starting with lactam **216**, the synthesis started with the *N*-boc protection of the amide. Initially, a conjugate addition was envisioned between an organometallic reagent of **217** and an unsaturated amide; however, all efforts toward this direction proved unsuccessful. The group hence decided to proceed by the means of polarity inversion, to which end the attempted alkyl-Suzuki coupling also failed. When trifluoroborate **217** was in turn subjected to photoredox nickel/iridium dual catalysis, 60% yield of the key coupled intermediate could be obtained. Mechanistically, the reaction is proposed to start by oxidative addition of **218** to Ni(0), and complex **221** then traps radical **220** generated from borate **217** by iridium photocatalysis. At this stage, nickel complex **222** is likely to undergo reductive elimination to release **219**, after which a SET between the iridium photocatalyst and nickel catalyst closes both catalytic cycles. With this key intermediate in hand, the rest of the synthesis was completed in 10 steps. To further demonstrate the importance of photocatalytic nickel cross-coupling reactions, it has also been employed as a key step in the formal syntheses of grandilodicine C (Zu, 2022) and lysergic acid (Opatz, 2024).^217,218^

Finally, the dual photoredox/cobalt catalysis has inspired numerous desaturation protocols over the years, among which, particularly, the advances toward acceptorless dehydrogenation are highly desired.^219–221^ However, cobalt photocatalysis is not solely limited to such desaturations, but the ability of cobalt to coordinate alkenes and carbonyls has led to the emergence of C–C bond-forming reactions.^222–225^ A dual cobalt/photoredox catalysis was employed in the conversion of a conjugated alkene into an unsaturated ketone as a part of the total synthesis of clionastatin A by the group of Zhang (Scheme 17C).^226^ Using testosterone as the starting material, intermediate **223** was then prepared in 13 steps. During the dual photocatalytic oxidation, the alkene was first oxidated to radical cation **225** by acridinium photocatalyst (see Section 2.3) to which water was added as a nucleophile. Upon deprotonation, the radical **227** was briefly generated before it went through desaturation by the cobalt complex; however, participation of the acridinium catalyst to this step has not been ruled out.^227^ Keto–enol tautomerization of **228** then gave **224**, which could be converted to the desired natural product in 2 additional steps.

### 2.12 Total Synthesis Driven by Photochemical Logic

Throughout this review, we have discussed the utilization of certain photochemical reactions to achieve key transformations in natural product synthesis. Alternatively, as a result of the vast method development over the past years, it has become possible to utilize photochemistry as a guiding light throughout almost every step in the synthesis of complex target molecules. As a testament to this, the group of Nicewicz published in 2025 their total synthesis of (−)-bisdehydrostemonine (Scheme 18).^228^ While stemona alkaloids have been a target for photochemistry-enabled syntheses before, the approach of Nicewicz managed to employ light-driven reactions in 6 of the 11 total synthetic steps.^229^ Commencing their endeavors, a photocatalytic hydroarylation of alkyne **229** with 2-bromopyridine **230** was carried out, giving vinylpyridine product **231** (Section 2.7). The alkene in **231** was then oxidized with the acridinium photocatalyst to a corresponding radical cation, which was attacked nucleophilically by acid **232** (Section 2.4). The remaining radical part of the oxidized alkene underwent an intramolecular 5-*exo*-trig cyclization, and after subsequent chloride elimination, yielded the lactone **233**. Olefination of **233** gave the exocyclic vinyl ether **234**, which again served to trap the radical formed from a single electron reduction of **235** (Section 2.7). The desired diastereomer of butanolide **237** was enriched by treating crude product **236** with TFA, followed by elimination of the methoxy substituent under basic conditions. The terminal alkene in **237** was then isomerized with the help of the decatungstate HAT-catalyst, resulting in a conjugated alkene in **238** (Section 2.8). Over the next two steps, the remaining seven-membered ring is closed, and the pyridine ring is contracted to the pyrrole in **239**. In the final stages, a decatungstate-catalyzed HAT is again harnessed to abstract the hydrogen atom on the aldehyde in **239**, paving the way to the addition of the final side chain via the Giese-coupling reaction with **240** (Section 2.8). From the (−)-bisdehydrostemonine A, the final natural product can be obtained by deoxygenative cyclization of the *in situ*-generation of the phosphoranyl radical (Section 2.8). In total, this photochemistry-driven total synthesis displays the shortest route to (−)-bisdehydrostemonine to date while simultaneously providing access to (−)-bisdehydrostemonine A.

**Scheme 18:**
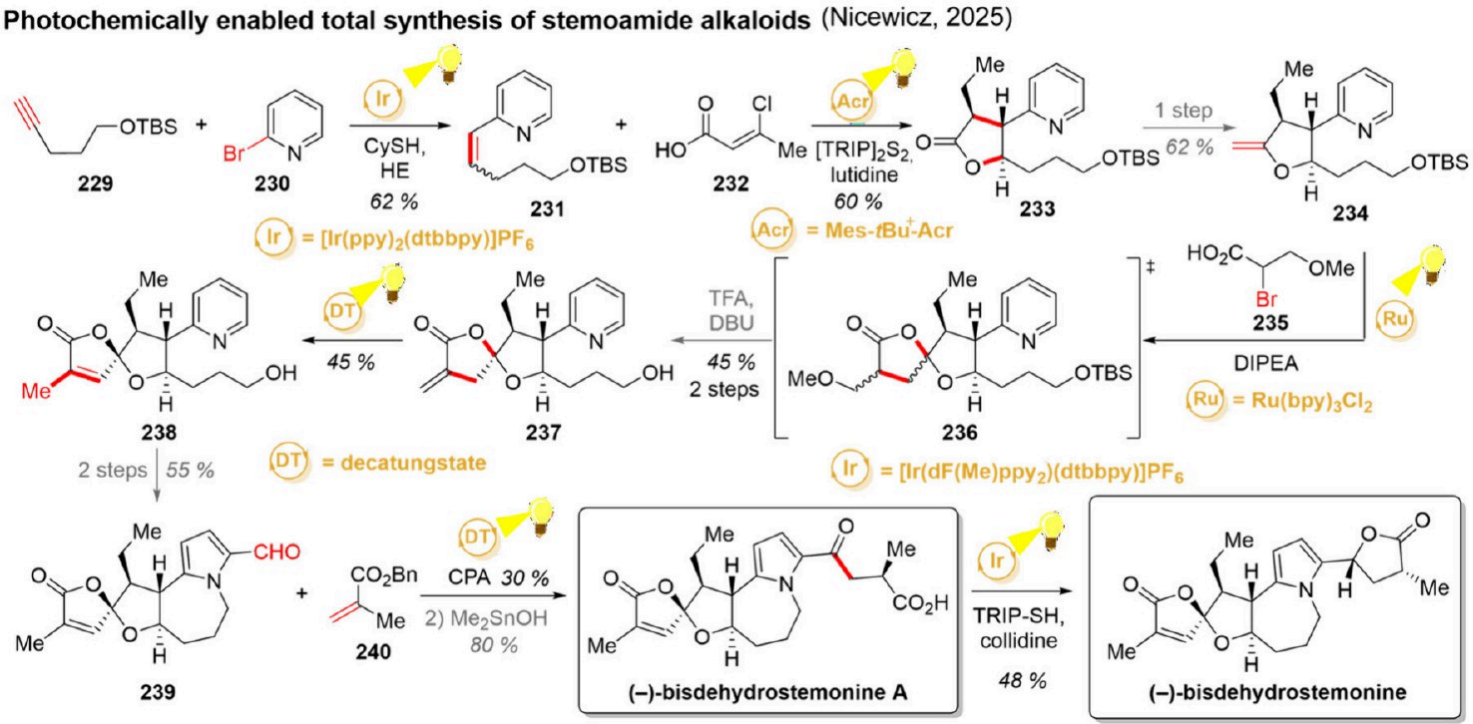
Total Synthesis of (−)-Bisdehydrostemonine Driven by Photochemical Synthesis Design

## 3 Summary and Outlook

In recent years, the use of photochemistry has established its role as a compelling tool for the synthesis of complex target molecules, fulfilling purposes ranging from generating molecular complexity to adjusting the peripheral functionalities. While approaches based on direct irradiation have been utilized for a long time, they still have retained their relevance, particularly in light-driven cyclizations ([2.2]-cycloaddition, photo-Diels–Alder, and photo-Nazarov reactions) and alkene isomerizations. To complement these transformations, photocatalyzed methods have also started to find their way into natural product synthesis. The possibility of activating substrates that themselves do not efficiently harvest light has profoundly broadened the applicability and versatility of photochemical methods available for synthetic chemists. This has led to the recent application of transformations such as radical decarboxylation, redox-active ester fragmentation, and dehalogenation in the field of total synthesis. As an emerging field, we expect the synergy between transition-metal catalysis and photocatalysis to enable and enhance cross-coupling reactions in the upcoming decades. Furthermore, we strongly believe that HAT-driven epimerization will be recognized as a revolutionary method for stereoediting, offering a complementary approach to invert stereocenters with hydridic instead of acidic protons. Despite these recent advances, the utilization of photocatalyst-driven transformations in natural product synthesis is still somewhat in its infancy, and many intriguing reactions, such as ligand-to-metal charge transfer (LMCT) protocols and halogen atom abstraction (XAT), have yet to be introduced into natural product chemistry.
